# The Rényi divergence enables accurate and precise cluster analysis for localization microscopy

**DOI:** 10.1093/bioinformatics/bty403

**Published:** 2018-06-01

**Authors:** Adela D Staszowska, Patrick Fox-Roberts, Liisa M Hirvonen, Christopher J Peddie, Lucy M Collinson, Gareth E Jones, Susan Cox

**Affiliations:** 1 Randall Centre for Cell and Molecular Biophysics, King’s College London, London, UK; 2 Electron Microscopy Science Technology Platform, The Francis Crick Institute, London, UK

## Abstract

**Motivation:**

Clustering analysis is a key technique for quantitatively characterizing structures in localization microscopy images. To build up accurate information about biological structures, it is critical that the quantification is both accurate (close to the ground truth) and precise (has small scatter and is reproducible).

**Results:**

Here, we describe how the Rényi divergence can be used for cluster radius measurements in localization microscopy data. We demonstrate that the Rényi divergence can operate with high levels of background and provides results which are more accurate than Ripley’s functions, Voronoi tesselation or DBSCAN.

**Availability and implementation:**

The data supporting this research and the software described are accessible at the following site: https://dx.doi.org/10.18742/RDM01-316. Correspondence and requests for materials should be addressed to the corresponding author.

**Supplementary information:**

Supplementary data are available at *Bioinformatics* online.

## 1 Introduction

Localization microscopy is a super-resolution imaging method based on detecting randomly activated, single molecules in a sequence of images. A super-resolution image is then reconstructed using this list of single molecule localizations (Betzig *et al.*, 2006; Rust *et al.*, 2006). A common application of localization microscopy is to study proteins clustered on the cell membrane. These clusters [approximated as Gaussian (Sengupta *et al.*, 2011; Shivanandan *et al.*, 2015) or Lorentzian (Pertsinidis *et al.*, 2013)] can be analysed with quantitative methods to provide measurement of their properties or functions. For example, analysis of protein clusters present in the cell plasma membrane revealed their role in cell signalling [LAT, LFA-1 and Src-family proteins clusters in T-cells (Owen *et al.*, 2010; Shannon *et al.*, 2015)], dynamics [GPI-anchored proteins in COS-7 cells (Sengupta *et al.*, 2011)] and their influence on the structural changes [sphingolipid ceramides in Jurkat, U2OS and HBMEC (Burgert *et al.*, 2017)]. However, analysing this type of data is often challenging, as the data often contains background localizations or multiple fluorophore re-appearances (Deschout *et al.*, 2014), which can make it difficult to identify clusters and/or lead to inaccurate size measurements.

The methods used for cluster analysis of localization microscopy images can be roughly divided into spatial statistical methods [such as Ripley’s functions (Ripley, 1976)] and density based algorithms [such as DBSCAN (Ester *et al.*, 1996) or SR-Tesseler (Levet *et al.*, 2015)]. DBSCAN uses a distance measure to aggregate data points into separate categories: clustered points and noise. SR-Tesseler method is based on creating the Voronoï diagrams to segment data points into separate ‘cells’. Both of these methods use a global definition of density to filter areas with higher point aggregation (Ester *et al.*, 1996; Levet *et al.*, 2015). However, it has been argued that obtaining the density parameter for localization microscopy data may be difficult (Ankers *et al.*, 1999; Steinbach *et al.*, 2003). Generally, the spatial statistical analysis methods are more universal and require less prior knowledge about the system than the density based algorithms (Deschout *et al.*, 2014).

Ripley’s K function is a widely used method which provides a measure of the difference between the data distribution and a uniform distribution. There are three versions of Ripley’s functions called K, L and H (Supplementary Note S1). The K and L functions are usually used to detect the presence of clustering in the data and the H function is used to calculate cluster size using the maximum of H(r) (Deschout *et al.*, 2014; Sengupta *et al.*, 2011; Shannon *et al.*, 2015). Recently, it has been noted that measuring the cluster radius by detecting the radius for which the Ripley’s H function is maximal can be biased (Kiskowski *et al.*, 2009; Lagache *et al.*, 2013) and that the origin of this bias has been reported to be related to density of the clusters in the dataset (Kiskowski *et al.*, 2009). This can lead to different bias in the measured cluster radius for different cluster densities. Approaches have been proposed to remove the bias from the cluster radius measurements, for example by measuring the cluster size as a half of the radius for which the first derivative of the Ripley’s H function has the value of –1 (Kiskowski *et al.*, 2009) or by using a constant correction coefficient (Lagache *et al.*, 2013; Supplementary Note S1 and Supplementary Fig. S1a and b).

However, these methods do not remove the cause of the bias which is intrinsically related to the method itself, as this method weights signal (clustered points) and noise (not clustered points) equally (Supplementary Note S2). Additionally bias removal, as proposed by these approaches, can lead to new inconsistencies in the cluster radius measurements. For example, the first derivative of Ripley’s H function is calculated numerically, thus the frequency with which it is calculated is going to affect the first derivative value and detecting a specific value will be either impossible or provide worse results than a traditional method (Supplementary Fig. S1a). The other approach using a constant coefficient to decrease the measured cluster radius can influence the performance of Ripley’s H function and its resistance to noise (Supplementary Fig. S1b). It has also been suggested that these methods for bias removal may be better suited for analysis of simulated datasets than real data (Kiskowski *et al.*, 2009).

Here, we propose use of the Rényi divergence (Rényi, 1965) as a measure for cluster analysis. The Rényi divergence quantifies the difference between two distributions:

(1) $$D_{\alpha }(p(x)||q(x))=\frac{1}{\alpha -1}\ln[\int_{x}p(x){\left(\frac{p(x)}{q(x)}\right)}^{\alpha -1}dx],$$

where *p*(*x*) is the data distribution and *q*(*x*) is a clustered reference distribution (if different than clustered reference distribution has been used it has been indexed, e.g. ‘U’ for the uniform distribution, Supplementary Note S3). We have used a clustered reference distribution as more closely related to the sample distribution and is therefore relevant for analysis clustered biological samples. Ripley’s H function can be derived from the Rényi divergence (for α = 2), and a uniform reference distribution $q^{U}(x_{i})=\frac{1}{A}$ (Supplementary Note S4). For the purpose of clustering analysis, we used a sampling approximation of the Rényi divergence:

(2) $$D_{\alpha }\propto \frac{1}{\alpha -1}\ln\left[\frac{1}{N}\sum_{i=1}^{N}{\left(\frac{1}{N}\right)}^{\alpha -1}{\left(\sum_{j=1}^{N}I(d_{ij}<r)\right)}^{\alpha -1}\right],$$

where *N* is the number of points in the dataset, α is the scaling coefficient, *d_ij_* is the distance between the *i*th central point and the *j*th point, and $I(d_{ij}<r)$ is an indicator function, which has value 1 for $\left(d_{ij}<r\right)$ and 0 otherwise. The scaling coefficient *α* controls the Rényi divergence function value, as it promotes areas with higher density (clusters) over the lower intensity of background noise (Supplementary Notes S3 and S2). It should be also noted that in the case of clustering analysis all points which belong to any cluster are treated as signal. The not clustered points we have called noise, these points originated from localizations of background fluorescence, free floating dye or non-specific staining. In this study we compare the performance of our method, based on the Rényi divergence, with Ripley’s H function, using simulated and experimental data. For experimental data we have also compared the performance of the Rényi divergence with SR-Tesseler and DBSCAN, which are the density based clustering methods. We have imaged three types of structures to assess the precision and accuracy of the Rényi divergence as a measure of cluster size: DNA-origami plates with known size (Rothemund, 2006), clathrin-coated pits in HeLa cells, the size of which has been confirmed via electron microscopy (Saffarian *et al.*, 2009; Sochacki *et al.*, 2017), vaccinia virus particles in HeLa cells, which were imaged with correlative localization and electron microscopy (Brama *et al.*, 2015; Peddie *et al.*, 2017) and podosome cores in THP-1 cells which size varies across the cell and were previously imaged using electron microscopy (Tanaka *et al.*, 2015) and atomic force microscopy (Labernadie *et al.*, 2010; Luxenburg *et al.*, 2007). These biological structures were selected because they can be observed in different cell types. Clathrin coated pits are responsible for active intra-cellular transport (endocytosis), the mechanism of which is similar across different cell types. The vaccinia virus is a poxvirus previously used in smallpox vaccination. Currently, the vaccinia virus is used for study of gene expression and for creating new vaccines [using a vaccinia virus mutant (Jacobs *et al.*, 2009)].

## 2 Materials and methods

### 2.1 Sample preparation

DNA origami is structures formed by artificial folding of DNA. Here, we used flat DNA origami plates with known size of 60 × 90 nm (GATTAquant). The DNA plates were uniformly covered with Alexa Fluor 647 molecules (each plate with approximately 20 molecules). Localization microscopy samples were prepared according to the protocol supplied by GATTAquant. The DNA origami was immobilized on BSA-biotin-neutravidin surface. The dishes (35 mm dishes with #1.5 glass coverslip bottom, Ibidi, Germany) were washed with PBS (three times) and incubated with 200 μl of BSA-biotin solution (0.5 g/l in PBS) for 5 min, followed by biotin solution removal and further washes in PBS (three times). Dishes were then incubated with neutravidin solution (0.5 g/l in PBS) for 5 min (neutravidin solution was removed and washed with PBS, three times). The diluted solution of DNA origami (around 100 times: 0.5 μl of DNA origami solution + 50 ml of PBS) was placed in the dish and incubated for 5 min followed by a washing step (three times in PBS). The optimal dilution factor was selected using guidelines provided in the sample preparation protocol, leading to an average density of DNA-origami plates ∼1 plate/${\mu m}^{2}$.

Samples with labelled clathrin coated pits were prepared using HeLa cells (ATCC, CCL-2) seeded on 35 mm dishes with #1.5 glass coverslip bottom (Ibidi, Germany) ∼5$\times 10^{4}$ cells/dish. After leaving the cells to adhere for 16 h, the cells were fixed for 20 min in 3.6% formaldehyde, permeabilized for 5 min in 1% saponin and blocked in 3% BSA for 30 min. The anti-clathrin anti-body (BD Biosciences, 610499) was added diluted 1:200 or 1:500 in PBS with 3% BSA and 0.5% saponin and incubated for 1 h. An Alexa Fluor 647 conjugated secondary anti-body (Invitrogen, A21235) was diluted to 1:500 in PBS with 3% BSA and incubated for 30 min.

Vaccinia virus samples were prepared according to the protocol published in (Peddie *et al.*, 2017).

Podosome samples were prepared using THP-1 cell line (ATCC, TIB-202). The cell culture, fixation and staining were performed according to the protocol presented in Staszowska *et al.* (2017); Vijayakumar *et al.* (2015). For the staining of actin present in the podosome core we have used Alexa Fluor 647 conjugated to phalloidin (Invitrogen, A22287). To identify podosomes in fixed cells, adhesive rings were confirmed using vinculin conjugated Alexa Fluor 488.

### 2.2 Imaging

The localization microscopy imaging was performed using the STORM system at Nikon Imaging Center at King’s College London. This system is equipped with an Eclipse Ti-E Inverted Nikon Microscope, Andor Ixon EMCCD back-illuminated camera (DU-897U-CSO-#BU), laser and LED light sources (laser wavelengths and powers are: 405 nm, 30 mW; 488 nm, 90 mW; 514 nm, 50 mW; 561 nm, 90 mW and 647 nm, 170 mW) and is operated with NIS Elements software with N-STORM module. The imaging was performed with TIRF, using a 100x, N.A. 1.49 objective.

The 647 nm laser power was adjusted during the acquisition to acquire similar number of counts in every frame (as far as possible). To improve the signal-to-noise (S/N) of the images the excitation light angle was adjusted to include only the fluorophores at a given optical plane and exclude background fluorescence. The imaging angle was adjusted for every imaged section and selected so that the image has the highest possible contrast.

Prior to imaging, the samples (DNA origami, clathrin coated pits and podosomes) were immersed in the imaging buffer. The base buffer was made with β-Mercaptoethylamine (MEA, Sigma Aldrich, 30070-50G) according to the recipe presented in (Nikon, 2015). For better stability of the Alexa Fluor 647, Cyclooctatetraene (COT, 98%, Sigma Aldrich, 138924-1G) dissolved in DMSO (Sigma Aldrich, 472301-1L-D) was added to the base buffer to a final concentration of 2 mM (Dave *et al.*, 2009).

In each imaging series about 6000 frames for DNA origami samples and 10 000 for clathrin-coated pits and podosomes were acquired at a rate of 30–50 frames per second.

Imaging of vaccinia virus samples, using scanning electron microscopy and localization microscopy was performed as discussed in (Brama *et al.*, 2015; Peddie *et al.*, 2014).

Super-resolution images were reconstructed with ThunderSTORM (Ovesný *et al.*, 2014) using single-emitter and multi-emitter settings. Molecule localizations were filtered to remove false localizations with FWHM sizes much smaller than the predicted PSF (110 nm for DNA-origami, clathrin coated pit and podosome samples and 90 nm for vaccinia virus particles). Additionally, drift was removed from the reconstructed images using the cross correlation option with binning 5 and magnification 5.0.

### 2.3 Monte Carlo data generation

The simulated datasets for cluster analysis were created using C++ and Matlab (2016b). For each dataset the cluster positions were selected randomly. Clusters were simulated with desired parameters varying the number of clusters in the dataset, density of points in the cluster, cluster size, distribution of points in the cluster (uniform or Gaussian). Uniform background noise was added at the desired level. Both the noise and clustered points had the same intensity. Signal-to-noise ratio was calculated for each noise level as a ratio of number of points in the same area, in areas classed as signal and background. See Supplementary Figure S2 for more information about signal-to-noise ratio and an example of the data.

### 2.4 Localization microscopy data simulation

The localization microscopy data were simulated using a Matlab (2016b) software. The positions of fluorophores in the data were defined using 10 datasets simulated for Monte Carlo testing (10 clusters with 80 and 160 nm radius). As the localization precision and quality of data depends in a great deal on the density of data we have simulated single molecules with varying density (by setting a different number of frames for which a fluorophore was in the dark state). For each density, 3000 frames of single molecule data were simulated with pixel size 100 nm. We have also set the simulated fluorophores not to bleach permanently. Then the single molecule images were analysed with ThunderSTORM (similarly to experimental data) and clustering analysis.

### 2.5 Clustering analysis software

The Rényi divergence analysis of clustering datasets was performed using Equation (14) in Supplementary Note S3. The Rényi divergence was calculated for a range of α values (usually 10–120 with increments of 10). Additionally, the same software also computed the Ripley’s H function. The first and second derivatives of the Rényi divergence and Ripley’s H function were calculated using the numerical derivative: $f\prime (x)=\frac{f(x+h)-f(x-h)}{2h}$ and $f\prime \prime (x)=\frac{f\prime (x+h)-f(x-h)}{2h}$ for second derivative calculation. The step size *h* was set to be equal to the sampling for the Rényi divergence and Ripley’s function calculation. It should be noted that the derivative calculated for the Rényi divergence clustering analysis was only used to detect gradient changes in the Rényi divergence and its first derivative curves.

Prior to the Rényi divergence and Ripley’s H function calculation for the clathrin coated pit and vaccinia virus particle datasets, we have selected regions of interest for the localization microscopy images. This was performed to include only areas where the analysed structures were present in the images, and exclude areas where clusters were not present or false clustering occurred. For clathrin coated pits we have created mask images to cover the area of the HeLa cell as the clathrin pits were present throughout the cell. Region of interest was selected similarly also for podosomes, to include only the areas where the podosomes were present in the images. For vaccinia virus particles we have selected rectangular regions of interest containing a high density of vaccinia virus particles. The masks and notes on the size of each region of interest can be found in the data bundle. It should be noted that no region of interest was selected for analysis of DNA-origami samples as the DNA-plates were randomly distributed across the whole image.

The density based clustering was performed using the SR-Tesseler software (Levet *et al.*, 2015), which also performed the DBSCAN analysis. The cluster radius was also calculated using SR-Tesseler software using the Voronoï diagrams, which segmented the data into single point domains. Then the segmented image was thresholded to exclude regions with density which was two times smaller than the average density [as suggested in (Levet *et al.*, 2015), we have also tested different values of the density threshold parameter, Supplementary Fig. S3]. The last step was to spatially filter the diagram image to exclude structures smaller or bigger than the size of the analysed structure. Both the SR-Tesseler and DBSCAN measured the cluster radius using an ellipse fitted to a segmented cluster patch. The cluster radius is the calculated as a half of an average of the longest and shortest axis of the fitted ellipse. It should be also noted that the fitted ellipses were smaller than the segmented clusters, leading to underestimation of cluster size. For DNA origami we set the desired cluster radius to be between 15 nm and 100 nm, for clathrin coated pits––50 nm and 170 nm and Vaccinia virus particles 60 nm and 170 nm. For the DBSCAN analysis we have adjusted the distance parameter, which is used by the software to estimate cluster radius: 54 nm for DNA origami (Supplementary Note S5), 100 nm for clathrin coated pits [estimated based on the electron microscopy images presented in (Sochacki *et al.*, 2017)] and 108 nm for vaccinia virus particles (measured for scanning electron microscopy images).

### 2.6 Cluster radius calculation

The cluster radius was calculated using the Rényi divergence and Ripley’s H function. The Rényi divergence for a specific α was calculated as a function of radius. The function counts the number of points inside a given radius. Because of the weighting effect of α, the main component of the function value comes from high density areas (clusters). Thus, when the radius is equal to the actual cluster size the function value does not change or changes by a very small amount for several radius values. This means that a plateau can be detected on the function and the cluster radius can be found by looking for points with gradient equal to 0 (first and second derivatives of function need to be equal to 0). In practice, we did not detect the 0 gradient but set a tolerance values for the first and second derivatives. We have used a tolerance of 1% change for the first and 0.5% for the second derivatives. These values were set experimentally, using simulated data with 10 clusters, 80 nm radius with and without any noise points. We have used the same tolerance values for all analysed data. In practice, we have used a set of tolerance values for the plateau detection. Our algorithm searched for the first point in a plateau as an estimate for the radius value (at least three points needed to be equal to 0 within a set tolerance).

The radius measurements using Ripley’s H function were calculated by detecting the maximum of the Ripley’s H function.

### 2.7 Estimation of size of clathrin coated pits

Clathrin coated pits have been extensively studied using electron microscopy (Saffarian *et al.*, 2009; Sochacki *et al.*, 2017), and their size in HeLa cells depends on the size of their cargo (Saffarian *et al.*, 2009). A visual inspection of electron microscopy images suggests a size of around 100 nm (Saffarian *et al.*, 2009; Sochacki *et al.*, 2017). Additionally, we also performed a visual check of the cluster radius measurement for the clathrin coated pits. We have used a localization microscopy image of the clathrin coated pits (prepared with 1:200 anti-clathrin anti-body and pre-processed with single-emitter fitting) to draw circles which enclosed the clustered points. We have selected this dataset at random (it was dataset six from the first sample). Each circle drawn was numbered (Supplementary Fig. S4c) and its radius measured (by saving the size of the drawn circle). We have collected results from 203 clusters and these are presented in Supplementary Figure S4d. The mean radius measured was 74 ± 13 nm, close to the expected value and radius measured with the Rényi divergence. We have also used the same methodology to investigate size of DNA origami in reconstructed localization microscopy images.

### 2.8 Detection and characterization of vaccinia virus particles in electron microscopy images

HeLa cells infected with vaccinia virus were prepared using an in-resin fluorescence protocol which introduces electron contrast into the sample whilst preserving fluorescence (Peddie *et al.*, 2014). Thus, the virus particles appear in the electron microscopy images as dark circular features, often surrounded by a bright ring. In order to match the area where the fluorescent proteins are present in the sample, we applied a thresholding method to include only electron dense core of each particle. The radius of each particle was measured as an average of its shortest and longest dimension. It should be also noted that in this method each particle radius is measured separately, while the Rényi divergence and Ripley’s H function provide an average measurement for a dataset.

The analysis of vaccinia virus particles was performed using Matlab (2016b). Each electron microscopy image was thresholded using a range of thresholds to account for the variation of brightness on the image (this was usually from 75% to 100% of the mean intensity value). Thresholded images were binarized using im2bn() function and inverted using function imcomplement() to ensure that the white areas in the binary images correspond to the areas with low intensity on the original image. Some particles had holes and empty edges due to variation in brightness in the original image, these were filled using imfill() and bwmorph(). Then, particles were filtered using a spatial filter (excluding particles smaller or bigger than a set range of area values, here it was 40 and 600 pixels, with pixel size 16.5 nm), eccentricity and solidity filter, which used a statistic provided by the regionprops() function. The filtering to remove elliptical and empty (non-solid) particles was performed using experimentally set ranges, 0–0.8 for elliptical filtering and 0.2–1 for solidity filtering. The particles identified by the software were confirmed by the user, by selecting the correct particle identifications. Lastly, the software saved an image with identified particles and a file containing information about the radius of each virus particle (see Supplementary Fig. S4 for a region of a particle identification image).

### 2.9 Statistical analysis

We used Monte Carlo simulations to establish the stability of our analysis method compared with Ripley’s H function. The Monte Carlo testing was performed for simulated data similarly to (Kiskowski *et al.*, 2009). Random datasets with the same characteristics (as an original dataset––number and size of clusters, noise level) were generated and for each new dataset cluster radius measurements with the Rényi divergence and Ripley’s H function were performed. The number of the Monte Carlo repetitions was selected by testing the changes in the value of measured mean and SD. The simulations stopped when the variation in both of these values was around 0.5% (Supplementary Fig. S5).

**Fig. 1.**
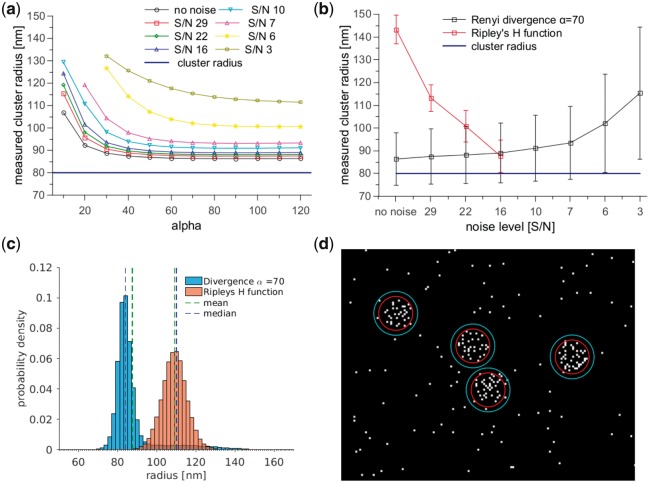
Variation of measured cluster radius with α and noise level for 100 000 simulated datasets. Clusters of radius 80 nm were simulated with a uniform distribution of fluorophores across the cluster. (**a**) Results of cluster radius measurements with different α values for datasets with increasing levels of noise (measured as mean). (**b**) Comparison of cluster radius values measured with the Rényi divergence (marked with black) and Ripley’s H function (red) for datasets with increasing noise levels. The Ripley’s H function radius measurements were only possible for datasets with S/N higher than 16 [for lower S/N H(r) function does not have a maximum]. The Rényi divergence provided stable radius measurements S/N as low as 6. (**c**) The histogram of results of cluster radius measurements for simulated clusters with S/N 29. The mean and median are shown in dashed lines for each method (in green and blue, respectively). (**d**) Visualization of the radius measured with the Rényi divergence (red circles) and Ripley’s H function (cyan circles) for simulated clusters with radius 80 nm and S/N 29

The histograms of the results of cluster radius measurements for simulated data suggested that the results do not belong to the same distributions (Fig. 1c). Thus to check if these results could originate from the same distribution we have performed the two-sample Kolmogorov–Smirnov test. Our null hypothesis was that they do belong to the same distribution. The test suggested that it is extremely unlikely that the results from the Rényi divergence and Ripley’s H function originate from the same distribution. The *P*-values for 5% confidence level are shown in Supplementary Table S1.

## 3 Results

### 3.1 Selection of the scaling coefficient α

The scaling coefficient α tunes the Rényi divergence response to different properties of data such as the cluster size/shape or the background noise level. The Rényi divergence calculation is performed by raising the number of points surrounding a central point (in a given radius) to a power (α−1). Thus, the Rényi divergence value from the regions with higher density (clusters) is greater than from those with smaller density of points (noise) for α>2 (Supplementary Note S3). This also means that the signal is promoted over the noise. The optimal value of α should be selected according to the data properties and usually higher noise levels require a higher value of α. In our investigations, we selected α by looking for no or small change in the measured cluster radius value (less than a 2% change as α is increased further, Fig. 1a). In this work, we used a single value of α = 70, as it provided stable radius measurements for all analysed datasets (for example, Fig. 1a). Additionally, we investigated the noise resistance of the Rényi divergence and Ripley’s H function on simulated datasets with different noise levels (Fig. 1b and Supplementary Fig. S1c–e).

The results for simulated data suggest that the Rényi divergence provides a more accurate and reliable cluster radius measurements than Ripley’s H function. We have analysed data with different size of clusters (80 nm and 160 nm radius, Fig. 1 and Supplementary Fig. S1c), different point distribution across the clusters (uniform and Gaussian, Supplementary Fig. S1), different number of points in the same size cluster (Supplementary Fig. S1), different number of clusters in a dataset (Supplementary Fig. S7). These datasets were simulated with uniform noise. We have also simulated single molecule data with different densities to test an impact of artefacts and localization precision on fidelity of cluster radius measurement (Fox-Roberts *et al.*, 2017) (Supplementary Figs S8 and S9). The results for different cluster types were similar so here we discuss one example, the uniform clusters with 80 nm radius (for results for other cluster types see Supplementary Figs S1 and S6–S9). The results for simulated data suggest that the Rényi divergence provides a more accurate and reliable cluster radius measurements than Ripley’s H function. The cluster radius measurements for data with different noise levels suggest that the Rényi divergence provides similar values of radius for different levels of noise caused by background localizations. For the same data Ripley’s H function can struggle to provide similar measurements or be unable to measure the cluster radius for data with high noise or small number of clusters (because the function does not have a maximum, required for the cluster measurement, Fig. 1b). The comparison of the SDs suggest that while the Rényi divergence scatter is higher than for Ripley’s H function, it remains similar only slightly increasing with noise (∼15 nm, Fig. 1b and c and Supplementary Fig. S1c–e).

It should be also noted that the cluster measured with the Rényi divergence has a small bias up to around 15%. We think that this is connected to the method used to measure clustering by detecting the gradient changes in the Rényi divergence curve. The formula used for the numerical derivative calculation increases the measured radius (offset by a sampling step value). Other cause of the biased cluster radius measurement is reporting of the mean value of the radius as it is more widely used statistical measure. As the Rényi divergence results display a sharp peak and a long tail the median indicates much better the most measure value (Fig. 1). For Ripley’s H function the SD was higher for datasets with no or low noise (∼5 nm for uniform and ∼30 nm for Gaussian clusters) and lower for higher noise levels (∼3–5 nm). This suggests that the accuracy of the measurement is less influenced by noise for the Rényi divergence than for Ripley’s H function.

### 3.2 Cluster size measurement for localization microscopy images

In addition to investigating performance of the Rényi divergence and Ripley’s H function with simulated data, we also used localization microscopy data of DNA origami, clathrin coated pits, vaccinia virus particles and podosomes. These structures were selected because we could confirm their size through techniques other than localization microscopy. For each structure, we have collected a series of images and localized single molecules using ThunderSTORM (Ovesný *et al.*, 2014) with single- and multi-emitter fitting (for multi-emitter data results see Supplementary Fig. S10).

The DNA origami constructs were designed to have a specific shape and size (Rothemund, 2006), here 60 × 90 nm rectangular plates (with around 20 Alexa Fluor 647 molecules per plate). As the Rényi divergence and Ripley’s H function are designed to detect circular clusters, the measured cluster size is not directly related to the plate size. We estimated that the measured cluster radius should be smaller than the radius of a circle passing through all corners (radius 54 nm) of the DNA-origami plate and bigger than a circle enclosed on the plate (radius 30 nm, Supplementary Fig. S4a and Supplementary Note S5). To validate this, we simulated rectangular plates with size and shape of the actual DNA plates. We have observed that the mean radius measured for the simulated rectangular plates was 52 ± 8 nm (around 4% smaller than the maximum expected radius) for the Rényi divergence measurement, and 69 ± 5 nm (22% bigger than the maximum expected radius) for Ripley’s H function. We expected to see this behaviour preserved in the experimental results.

**Fig. 2.**
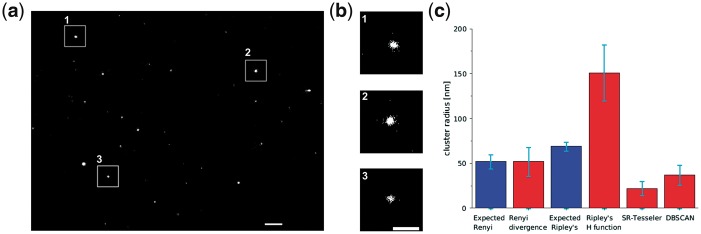
Measurement of the average size of the DNA-origami plates in localization microscopy data. (**a**) Localization microscopy image of DNA-origami plates, analysis with single-emitter fitting. Scale bar 1 μm. (**b**) Magnified images of single DNA-origami plates. Scale bar 500 nm. (**c**) The average expected radius based on the simulations (50 000 repetitions) and the radius measured in the experimental data with the Rényi divergence (52 ± 8 nm for simulated and 52 ± 16 nm for experimental data), Ripley’s H function (69 ± 5 nm and 151 ± 30 nm), SR-Tesseler (22 ± 8 nm) and DBSCAN (37 ± 11 nm). The error bars are the SD

We analysed localization microscopy datasets collected from five DNA origami samples analysed using single-emitter fitting (for multi-emitter fitting results see Supplementary Fig. S10a). The results suggest that the Rényi divergence provides cluster radius measurements which are more accurate than Ripley’s H function (Fig. 2c). While the average cluster radius measured with the Rényi divergence was equal to the expected radius (52 ± 16 nm), the cluster radius measured with the Ripley’s H function was almost three times bigger (151 ± 32 nm). Moreover, the comparison of scatter for the Rényi divergence (SD 16 nm) and Ripley’s H function (SD 32 nm) suggests that the Rényi divergence is more precise. The experimental DNA origami data were also analysed with density based clustering methods: SR-Tesseler and DBSCAN. DBSCAN measured cluster radius which was exactly equal to an average of the plate size, when SR-Tesseler measured smaller cluster radius (22 ± 8 nm for SR-Tesseler and 37 ± 11 nm for DBSCAN, Supplementary Note S5).

We have also measured sizes of the clathrin coated pits present in HeLa cells. Clathrin-coated pits have been extensively studied using electron microscopy (Saffarian *et al.*, 2009; Sochacki *et al.*, 2017), and their size in HeLa cells depends on the size of their cargo (Saffarian *et al.*, 2009). A visual inspection of electron microscopy images suggests a size of around 100 nm (Saffarian *et al.*, 2009; Sochacki *et al.*, 2017). We have imaged samples with two concentrations of anti-clathrin anti-body: 1:200 and 1:500 (three samples for each primary anti-body concentration, see Methods Section and Supplementary Fig. S10b and c), to establish if different amount of signal from the investigated structure affects the measured radius. For each labelling density, we have imaged 3 samples and selected at least 10 regions for imaging.

**Fig. 3.**
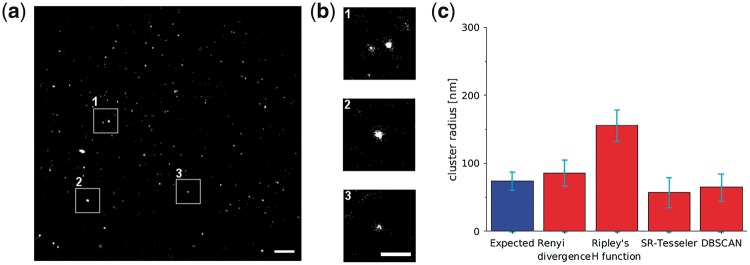
Measurement of the average size of the clathrin coated pits in localization microscopy data. (**a**) Localization microscopy image of clathrin coated pits in HeLa cells (labelling 1:200). (**b**) Magnified images of clathrin coated pits. Scale bar 500 nm. (**c**) The average expected (74 ± 13 nm) and measured radius, 86 ± 19 nm for the Rényi divergence and 156 ± 23 nm for Ripley’s H function. SR-Tesseler and DBSCAN measured cluster radius to be smaller than the expected value, 57 ± 22 nm and 65 ± 20 nm, respectively. Localization position fitting was performed with single-emitter fitting. The error bars are the SD

Thus, the accuracy of the radius measurements could be compared both with regard to how close the measured value is to the predicted value and how large the scatter in the measurements is, for the two labelling densities and different fluorophore localization methods. Our results suggest that the Rényi divergence provided a measurement which was both more closely related to the expected radius of the clathrin pits and more stable. The average radius measured with the Ripley’s H function, 156 ± 23 nm, was almost two times bigger than the radius measured with the Rényi divergence, 86 ± 19 nm. SR-Tesseler and DBSCAN measured cluster radii which were smaller than the expected cluster radius, 57 ± 22 nm and 65 ± 20 nm, respectively. We have also observed some variation in the values measured with regard to the labelling density for Ripley’s H function (see Results Section in Fig. 3b and Supplementary Fig. S10d–f). The Rényi divergence, SR-Tesseler, and DBSCAN measured radius values were not affected by the different labelling densities. Additionally, to confirm our cluster radius measurements for the clathrin coated pits we have performed a visual estimation of the pit size using a localization microscopy image (Methods Section and Supplementary Fig. S4c). The mean of the estimated radius was 74 ± 13 nm (Supplementary Fig. S4d), close to the expected value and radius measured with the Rényi divergence.

However, due to the limitations of any one imaging technique it is highly desirable to cross-validate results against a second imaging technique. Recent advances have made possible imaging of the same sample with simultaneous correlative light and electron microscopy (CLEM; Liv *et al.*, 2013; Zonnevylle *et al.*, 2013). Thus we could use the electron microscopy image as an estimation of ground truth and compare it to the quantitative results obtained from localization microscopy data.

**Fig. 4.**
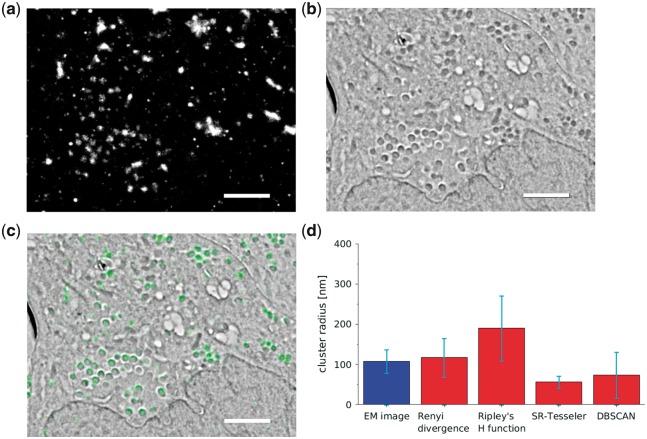
Vaccinia virus particles in HeLa cells imaging and radius measurements. (**a**) Localization microscopy image, and (**b**) electron microscopy image. (**c**) Overlay with electron microscopy image presented in grey-scale, and localization microscopy image in green. Scale bars 1 μm. (**d**) The average particle radius measured for electron microscopy (EM) images, 108 ± 29 nm and localization microscopy with the Rényi divergence (117 ± 48 nm) Ripley’s H function (190 ± 81 nm), SR-Tesseler (56 ± 15 nm) and DBSCAN (74 ± 57 nm). The error bars are the SD (Color version of this figure is available at *Bioinformatics* online.)

Vaccinia virus particles in HeLa cells were imaged using correlative localization and scanning electron microscopy. The samples were transfected with YFP-A3, which localizes to the core of viral particles. The fixed and frozen samples were then embedded in resin and cut into 200 nm thick sections (Brama *et al.*, 2015; Peddie *et al.*, 2017). YFP blinks in a partially hydrated state and so it was possible to obtain localization microscopy data by imaging at 200 Pa (Peddie *et al.*, 2017). Images of the matching areas in the sample were collected with localization and electron microscopy (Fig. 4a and b). Thus, the sizes measured for both of these imaging techniques could be directly compared for exactly the same structures. To estimate the true size of the vaccinia virus particles we analysed the electron microscopy images using an algorithm, which identified and characterized individual virus particles (Methods Section and Supplementary Fig. S4e–g). The mean radius of the virus particles in the electron microscopy images was 108 ± 29 nm. We compared this value with the average radius measured for localization microscopy data pre-analysed with single-emitter (Fig. 4d) and multi-emitter fitting (Supplementary Fig. S10g).

Analysis of vaccinia virus particle localization microscopy suggests that the Rényi divergence provides more accurate measurement than Ripley’s H function, SR-Tesseler or DBSCAN. For both single and multi-emitter fitting, the Rényi divergence provided cluster radius measurements, 117 ± 48 nm and 137 ± 62 nm respectively, which were very close to the particle radius measured in the electron microscopy images. However, the radius measured by Ripley’s H function, 190 ± 81 nm for single- and 221 ± 135 nm for multi-emitter fitting, was much bigger than the expected radius of virus particle. Values smaller than expected radius were measured for SR-Tesseler 56 ± 15 nm for single- and 58 ± 17 nm for multi-emitter fitting and for DBSCAN ±57 nm for single- and ±61 nm for multi-emitter fitting. It should also be noted that the SD values (error bars in Fig. 4 and Supplementary Fig. S10) for the Rényi divergence were smaller (48 nm for single-emitter and 62 nm for multi-emitter fitting) than for the Ripley’s H function (81 nm for single- and 135 nm for multi-emitter fitting) which suggests that our method provided a more consistent cluster radius measurement. DBSCAN provided similar level of precision to the Rényi divergence (57 nm for single- and 61 nm for multi-emitter fitting), where SR-Tesseler had the smallest SD for these datasets (15 nm for single- and 17 nm for multi-emitter fitting), however it also underestimated the size of the clusters by 42%.

**Fig. 5.**
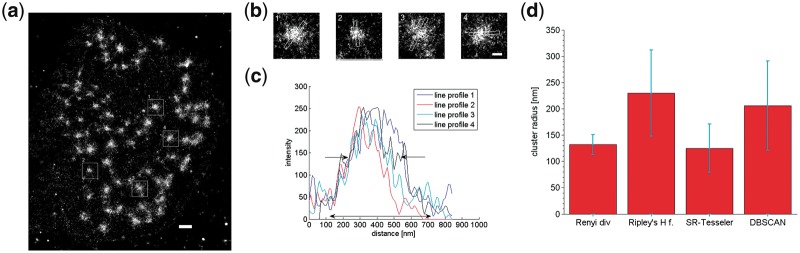
Podosome cores in THP-1 cells and their size measurement. (**a**) Localization microscopy image reconstructed with single emitter-fitting. F-actin podosome cores were stained with Alexa Fluor 647 conjugated to phalloidin (reconstructed with single emitter-fitting). Scale bar 1 μm. (**b**) Magnified single podosome cores with varying sizes. Scale bar 250 nm. (**c**) Line profiles of four podosome cores from (b). The podosome core has diameter of 500 nm at the base (indicated with black arrow) and between 200 to 300 nm in the most dense region of the core (indicated with two black arrows). (**d**) Cluster radius measurement of the podosome data. The Rényi divergence measured cluster radius of 132 ± 19 nm, Ripley’s H function 231 ± 82 nm, SR-Tesseler 125 ± 46 nm and DBSCAN 205 ± 85 nm. The error bars are the SD

Biological structures analysed above have very similar sizes through the cell, but this is not always the case in many biological structures. An example of such size variability is observed for podosomes. Podosomes are adhesive, actin rich structures formed by the cells to facilitate cell adhesion and migration (Schachtner *et al.*, 2013). They consist of two major components, an f-actin rich core and the ring complex (build with integrins and integrin associated proteins; Foxall *et al.*, 2016; Labernadie *et al.*, 2010; Tanaka *et al.*, 2015). The ring has diameter between 0.5 μm to 2 μm in diameter and 0.6 μm to 1 μm in depth (Meddens *et al.*, 2014; Rafiq *et al.*, 2016; Walde *et al.*, 2014). The core is smaller, usually around 0.5 μm in diameter (at the widest point) and 0.5 μm in height (Meddens *et al.*, 2014; Rafiq *et al.*, 2016). The size of podosomes varies across the cell, depending on their function or lifetime [for example, in migrating cells, the newly formed podosomes are larger than older ones (Meddens *et al.*, 2014)]. In reconstructed localization microscopy images (Fig. 5a and b), we have indeed seen a variation in the size of the podosome cores. It should be also noted that the podosome core has a shape of a cone, meaning that the localization density will be vary across the structure (the highest in the centre and lower on the edges). This has been confirmed using independent imaging techniques in (Labernadie *et al.*, 2010; Rafiq *et al.*, 2016).

Cluster radius measurements for podosome cores are less conclusive than cluster radius measurements performed for structures previously discussed in this paper. The podosome core size had been reported to differ across the cell and to be around 500 nm in diameter at the widest point (Labernadie *et al.*, 2010; Rafiq *et al.*, 2016). The podosome core has a cone shape, meaning that the density of localizations changes across the core with higher density in the centre and smaller density on the edge (Fig. 4c). The Rényi divergence and SR-Tesseler detected the radius the area of the core which has the highest density of localizations, 132 ± 19 nm and 125 ± 46 nm respectively. Both Ripley’s H function and DBSCAN reported a larger cluster radius, 231 ± 82 nm and 205 ± 85 nm. However, it should be noted that Ripley’s H function measured cluster radius only for 17 datasets out of 30, and for the rest it did not detect any clustering. The remaining methods did not have any problem with detecting clustering. The Rényi divergence provided a more stable cluster radius measurement with the smallest SD, than other methods. It also had similar SD for the measurement for data with clusters with the same size. While the SD for the remaining methods, Ripley’s H function, SR-Tesseler and DBSCAN, was higher than for the datasets with the same cluster size. Here, we discussed results for single-fitting, the results for multi-emitter fitting are presented in Supplementary Table S2. The results for multi-emitter fitting are similar to single-emitter results presented here, see Supplementary Table S2. We think that the results for podosome cores clearly highlight the property of the Rényi divergence to reliably detect areas with high point density.

The Rényi divergence has an additional advantage of requiring no user input. In particular, the values obtained with Ripley’s H function can depend strongly on the region selected for analysis (because of its equal weighting of signal and noise), whereas the Rényi divergence remains stable. This is important because it is common to restrict analysis to structures of interest to decrease the data volume. For example, for analysis of clathrin coated pits we have selected regions of interest to include the whole cell and exclude the almost empty area around it. We calculated the mean cluster radius for clathrin coated pits data with a selected region of interest (termed ROI) and for the whole field of view (termed Image). The mean radius measured for the data changes visibly for the Ripley’s H function (Supplementary Fig. S11). For the Rényi divergence it either changes by a small amount (Supplementary Fig. S11c) or remains the same (Supplementary Fig. S11a, b and d). This suggests that the Rényi divergence results are more universal and can be compared between different regions of interest (Supplementary Table S2).

The major drawback of the current form of the Rényi divergence is that it is set up to mainly analyse circular clusters. It is however possible to change the cluster shape detected by this method using similar methodology as presented for Ripley’s functions (Peters *et al.*, 2017). However, it should be also noted that the cluster size measurement can be still performed for small clusters with non-circular shape, as for example rectangular DNA origami plates. As the DNA origami plates used in this work were seen in reconstructed localization microscopy images as circular clusters due to limited localization precision.

## 4 Discussion

Localization microscopy data are more and more commonly used for quantitative analysis. However, measuring the exact size of the structures in the images/data can be problematic using the popular Ripley’s H function. Here, we have presented a method based on the Rényi divergence which can be tuned in response to noise and/or data properties. Our method provides more accurate cluster radius measurements than widely used Ripley’s H function. It also helps to overcome the bias present in the radius measurements provided by the Ripley’s H function. Similarly, when compared with the density based clustering methods, the Rényi divergence provides cluster radius measurement which is closer to the expected cluster radius. We think that the radius measurement was lowered for the density based methods, as they perform cluster radius measurement based on an ellipse fitted to a segmented cluster, which is smaller than the segmented cluster.

The Rényi divergence provides an accurate and precise method of cluster radius measurement. We have shown that the commonly used Ripley’s H function provides cluster radius measurements bigger (by around 60%) than the actual cluster radius, similarly to the results presented in (Kiskowski *et al.*, 2009; Lagache *et al.*, 2013). The Rényi divergence does not require user input or selection of region of interest (contrary to Ripley’s H function, Supplementary Fig. S11, SR-Tesseler or DBSCAN) and the selection of α can be done after the analysis was performed. Apart from these benefits the Rényi divergence calculation can be performed very fast, taking around 20 s to analyse a dataset containing 30 000 localized points.

## Supplementary Material

Supplementary Information

## References

[bty403-B1] Ankers M. et al (1999) OPTICS: ordering points to identify the clustering structure. Proc. ACM SIGMOD'99 Int. Conf. on Management of Data, 28, 49–60.

[bty403-B2] Betzig E. et al (2006) Imaging intracellular fluorescent proteins at nanometer resolution. Science, 313, 1642–1645. 16902090 10.1126/science.1127344

[bty403-B3] Brama E. et al (2015) Standard fluorescent proteins as dual-modality probes for correlative experiments in an integrated light and electron microscope. J. Chem. Biol., 8, 179–188.

[bty403-B4] Burgert A. et al (2017) Characterization of plasma membrane ceramides by super-resolution microscopy. Angew. Chem., 129, 6227–6231. 10.1002/anie.201700570 PMC5549273 28379629

[bty403-B5] Dave R. et al (2009) Mitigating unwanted photophysical processes for improved single-molecule fluorescence imaging. Biophys. J., 96, 2371–2381. 19289062 10.1016/j.bpj.2008.11.061 PMC2907709

[bty403-B6] Deschout H. et al (2014) Progress in quantitative single-molecule localization microscopy. Histochem. Cell Biol., 142, 5–17. 24748502 10.1007/s00418-014-1217-y PMC4072926

[bty403-B7] Ester M et al (1996) A density-based algorithm for discovering clusters in large spatial databases with noise In: Simoudis E. et al (eds) Proceedings of the Second International Conference on Knowledge Discovery and Data Mining (KDD-96), AAAI press, Paolo Alto, CA, pp. 226–231.

[bty403-B8] Fox-Roberts P. et al (2017) Local dimensionality determines imaging speed in localization microscopy. Nat. Commun., 8, 13558. 28079054 10.1038/ncomms13558 PMC5241698

[bty403-B9] Foxall E. et al (2016) Significance of kinase activity in the dynamic invadosome. Eur. J. Cell Biol., 95, 483–492. 27465307 10.1016/j.ejcb.2016.07.002

[bty403-B12] Jacobs B.L. et al (2009) Vaccinia virus vaccines: past, present and future. Antiviral Res., 84, 1–13. 19563829 10.1016/j.antiviral.2009.06.006 PMC2742674

[bty403-B13] Kiskowski M.A. et al (2009) On the use of Ripley’s K-function and its derivatives to analyze domain size. Biophys. J., 97, 1095–1103. 19686657 10.1016/j.bpj.2009.05.039 PMC2726315

[bty403-B14] Labernadie A. et al (2010) Dynamics of podosome stiffness revealed by atomic force microscopy. Proc. Natl. Acad. Sci. USA, 107, 21016–21021. 21081699 10.1073/pnas.1007835107 PMC3000246

[bty403-B15] Lagache T. et al (2013) Analysis of the spatial organization of molecules with robust statistics. PLoS One, 8, e80914–e80917. 24349021 10.1371/journal.pone.0080914 PMC3857798

[bty403-B16] Levet F. et al (2015) SR-Tesseler: a method to segment and quantify localization-based super-resolution microscopy data. Nat. Methods, 12, 1065–1071. 26344046 10.1038/nmeth.3579

[bty403-B17] Liv N. et al (2013) Simultaneous correlative scanning electron and high-NA fluorescence microscopy. PLoS One, 8, e55707. 23409024 10.1371/journal.pone.0055707 PMC3568124

[bty403-B18] Luxenburg C. et al (2007) The architecture of the adhesive apparatus of cultured osteoclasts: from podosome formation to sealing zone assembly. PLoS One, 2, e179. 17264882 10.1371/journal.pone.0000179 PMC1779809

[bty403-B19] Meddens M.B.M. et al (2014) Podosomes revealed by advanced bioimaging: what did we learn? Eur. J. Cell Biol., 93, 380–387. 25454791 10.1016/j.ejcb.2014.09.002

[bty403-B20] Nikon (2015) *Super Resolution Microscope N-STORM. STORM Protocol-Sample Preparation. (*10 November 2015, date last accessed).

[bty403-B21] Ovesný M. et al (2014) ThunderSTORM: a comprehensive ImageJ plug-in for PALM and STORM data analysis and super-resolution imaging. Bioinformatics, 30, 2389. 24771516 10.1093/bioinformatics/btu202 PMC4207427

[bty403-B22] Owen D.M. et al (2010) PALM imaging and cluster analysis of protein heterogeneity at the cell surface. J. Biophotonics, 3, 446–454. 20148419 10.1002/jbio.200900089

[bty403-B23] Peddie C.J. et al (2014) Correlative and integrated light and electron microscopy of in-resin GFP fluorescence, used to localise diacylglycerol in mammalian cells. Ultramicroscopy, 143, 3–14. 24637200 10.1016/j.ultramic.2014.02.001 PMC4045205

[bty403-B24] Peddie C.J. et al (2017) Correlative super-resolution fluorescence and electron microscopy using conventional fluorescent proteins in vacuo. J. Struct. Biol., 199, 120–131. 28576556 10.1016/j.jsb.2017.05.013 PMC5531056

[bty403-B25] Pertsinidis A. et al (2013) Ultrahigh-resolution imaging reveals formation of neuronal SNARE/Munc18 complexes in situ. Proc. Natl. Acad. Sci. USA, 110, 2. E2812–E2820. 10.1073/pnas.1310654110 PMC3725074 23821748

[bty403-B26] Peters R. et al (2017) Quantification of fibrous spatial point patterns from single-molecule localization microscopy (SMLM) data. Bioinformatics, 33, 1703–1711. 28108449 10.1093/bioinformatics/btx026

[bty403-B27] Rafiq N.B.M. et al (2016) Podosome assembly is controlled by the GTPase ARF1 and its nucleotide exchange factor ARNO. J. Cell Biol., 216, 181–197. 28007915 10.1083/jcb.201605104 PMC5223603

[bty403-B28] Rényi A. (1965) On the foundations of information theory. Revue de l’institut International de Statistique/Rev. Int. Stat. Inst., 33, 1–14.

[bty403-B29] Ripley B.D. (1976) The second-order analysis of stationary point processes. J. Appl. Prob., 13, 255–266.

[bty403-B30] Rothemund P.W.K. (2006) Folding DNA to create nanoscale shapes and patterns. Nature, 440, 297–302. 16541064 10.1038/nature04586

[bty403-B31] Rust M.J. et al (2006) Sub-diffraction-limit imaging by stochastic optical reconstruction microscopy (STORM). Nat. Methods, 3, 793–795. 16896339 10.1038/nmeth929 PMC2700296

[bty403-B32] Saffarian S. et al (2009) Distinct dynamics of endocytic Clathrin-coated pits and coated plaques. PLoS Biol., 7, e1000191–e1000118. 19809571 10.1371/journal.pbio.1000191 PMC2731173

[bty403-B34] Schachtner H. et al (2013) Podosomes in adhesion, migration, mechanosensing and matrix remodeling. Cytoskeleton, 70, 572–589. 23804547 10.1002/cm.21119

[bty403-B35] Sengupta P. et al (2011) Probing protein heterogeneity in the plasma membrane using PALM and pair correlation analysis. Nat. Methods, 8, 969–975. 21926998 10.1038/nmeth.1704 PMC3400087

[bty403-B36] Shannon M.J. et al (2015) Protein clustering and spatial organization in T-cells. Biochem. Soc. Trans., 43, 315–321. 26009169 10.1042/BST20140316

[bty403-B37] Shivanandan A. et al (2015) Accounting for limited detection efficiency and localization precision in cluster analysis in single molecule localization microscopy. PLoS One, 10, e0118767. 25794150 10.1371/journal.pone.0118767 PMC4368834

[bty403-B38] Sochacki K.A. et al (2017) Endocytic proteins are partitioned at the edge of the clathrin lattice in mammalian cells. Nat. Cell Biol., 19, 352–361. 28346440 10.1038/ncb3498 PMC7509982

[bty403-B39] Staszowska A.D. et al (2017) Investigation of podosome ring protein arrangement using localization microscopy images. Image Processing for Biologists. Methods, 115, 9–16. 27840289 10.1016/j.ymeth.2016.11.005

[bty403-B40] Steinbach M. et al (2003) Introduction to Data Mining. Springer, New York.

[bty403-B41] Tanaka H. et al (2015) Electron microscopic examination of podosomes induced by phorbol 12, 13 dibutyrate on the surface of A7r5 cells. J. Pharmacol. Sci., 128, 78–82. 25986486 10.1016/j.jphs.2015.03.002

[bty403-B42] Vijayakumar V. et al (2015) Tyrosine phosphorylation of WIP releases bound WASP and impairs podosome assembly in macrophages. J. Cell Sci., 128, 251–265. 25413351 10.1242/jcs.154880 PMC4294773

[bty403-B43] Walde M. et al (2014) Vinculin binding angle in podosomes revealed by high resolution microscopy. PLoS One, 9, e88251. 24523880 10.1371/journal.pone.0088251 PMC3921150

[bty403-B44] Zonnevylle A.C. et al (2013) Integration of a high-NA light microscope in a scanning electron microscope. J. Microscopy, 252, 58–70. 10.1111/jmi.12071 23889193

