# Supplementary material for: The Rényi divergence enables accurate and precise cluster analysis for localization microscopy
Source: Bioinformatics. 2018 Jun 1;34(23):4102–11. doi: 10.1093/bioinformatics/bty403 (PMC6247934; doi:10.1093/bioinformatics/bty403)
Supplement: Supplementary Information [file bty403_supplementary_information.pdf]

Supplementary information

1 Supplementary Note: Ripley's K function

Ripley's K function quantifies difference between two distribution (data and reference). It is the ratio of the number of points surrounding a central point within a given radius to the average point density in the image ($\frac{1}{A}$):

$$K(r) = \frac{A}{N^2}\left[\sum_{i=1}^{N}\sum_{j=1}^{N} I(d_{ij} < r)\right], \tag{3}$$

where A is the area of the image and N is the number of points on the image (29). The K function is usually normalised to a more useful form called L(r) which has a linear expected value for randomly distributed points:

$$L(r) = \sqrt{K(r)/\pi}. \tag{4}$$

Presence or absence of clustering can be deduced by comparing value of the L(r) function to linear expected value. For a random sample the L(r) function is equal or smaller than the expected value. If the value of L(r) is higher than the expected value the function is detecting clustering in the data. The L(r) function can be normalised to a function called H(r) which has an expected value equal to 0 for randomly distributed points:

$$H(r) = L(r) - r. \tag{5}$$

Ripley's functions can be used to detect the presence (6; 13; 11; 33) or degree of clustering when comparing between different conditions (6; 10). The H function can also be used to measure cluster radius (13).

Cluster size measurements using the maximum of Ripley's H function have been reported to exhibit bias. It has been suggested that the radius for which the Ripley's H function has a maximum is connected to the cluster separation rather than to the cluster size itself (13). It has been suggested that finding half the radius for which the first derivative of Ripley's H function is equal to -1 will find the cluster radius more accurately. An other approach has been suggested in (15), where a bias was identified directly in the Ripley's H function and corrected with a constant coefficient (the radius was identified as the radius for the maximum of Ripley's H function and divided by 1.3). It should be noted, however, that use of these approaches may introduce new problems. For example, detecting a specific value in a numerically calculated function is going to be affected by the sampling ratio used (see Supplementary Fig. 1a). Additionally, it has been found that this method can perform much worse than just detecting the maximum for experimental data (13). Lastly, use of a constant coefficient to calculate the cluster radius can be problematic especially for smaller clusters, causing detection of cluster radius to be smaller that it actually is (see Supplementary Fig. 1b).

2 Supplementary Note: The importance of α for noise resistance

The α value controls the response of the Rényi divergence to noise in the data. Both the Rényi divergence and Ripley's functions estimate the data by counting how many points surround every point in the data in a given radius. For the Rényi divergence any α can be used. For this study we have selected α=70, meaning that the number of points in a given radius was taken to the power (α-1)=69. Ripley's functions are a special case of the Rényi divergence with α=2. The significance of this becomes clear if we consider the contribution to the final value of the function from a noisy and clustered regions. If we select regions of radius 80 nm from a simulated dataset (see Supplementary Fig. 2a), one of which contains only noise (6 points) and one a whole cluster (35 points). For the Rényi divergence the value from noisy region is $6^{70-1} = 4.92 \times 10^{53}$ and $35^{70-1} = 3.47 \times 10^{106}$ from the cluster (difference $\sim 10^{53}$). For the same region Ripley's K functions measures $6^{2-1} = 6$ and $35^{2-1} = 35$ (difference 29). While both the Rényi divergence and Ripley's H function detect the difference between the noise and clustered regions, the difference measured with the Rényi divergence is far greater than for the Ripley's H function.

3 Supplementary Note: The Rényi divergence and its properties

The Rényi divergence is defined as:

$$D_\alpha(p(x)||q(x)) = \frac{1}{\alpha - 1} \ln \left[\int_x p(x) \left(\frac{p(x)}{q(x)} \right)^{\alpha - 1} dx \right] \tag{6}$$

where x is a continuous variable, $p(x)$ and $q(x)$ are probability density functions over x. It has following properties:

1. The Rényi divergence is non-negative for all distributions $p(x_i)$, $q(x_i)$, and for α bigger than 0: $D_\alpha(p||q) \geq 0, \forall_{p,q,\alpha>0}$
2. The Rényi divergence is equal to 0 only when distributions $p(x_i)$ and $q(x_i)$ are equal: $D_\alpha(p||q) = 0$ if $p(x) = q(x) \forall_{x \in \mathbb{R}}$
3. The Rényi divergence for $\alpha = 1$ is equal to Kullback-Liebler divergence (D_{KL}):
$\lim_{\alpha \to 1} D_\alpha(p||q) = D_{KL}(p||q)$.

For the purpose of clustering analysis of discrete single molecule localisation data we use sampling approximation of equation (6)

$$D_\alpha(p(x)||q(x)) \approx \frac{1}{\alpha - 1} \ln \left[\frac{1}{N} \sum_{i=1}^{N} \left(\frac{p(x)}{q(x)} \right)^{\alpha - 1} \right] \tag{7}$$

where the data set is defined as $X = \{x_1, x_2, ..., x_N\}$ and the probability distribution $p(x)$ is defined using a Parzen window estimation:

$$p(x_i) = \frac{1}{N} \sum_{j=1}^{N} \frac{I(d_{ij} < r)}{\pi r^2}, \tag{8}$$

where N is the number of data points, d_{ij} is the distance between points i and j, r is the radius around the central point, and $I(d_{ij} < r)$ is an indicator function (it has value 1 when two point are separated by distance smaller than the set value of r, and 0 when they are separated by a larger distance). Here, the function also counts the centre point to the number of points inside a given radius, which guarantees robustness to outliers. This means that the calculation of the Rényi divergence and Ripley's function will be biased for small values of radius. The value of $p(x_i)$ for a small radius when the centre point is not counted is 0, and when it is counted $p(x_i) = \frac{1}{\pi r^2}$. This bias is reduced by the logarithm function in the divergence. It can also be reduced by changing the sampling rate.

The Rényi divergence also requires a choice of a reference distribution, $q(x)$. Our choice of $q(x)$ is non-standard, we selected a distribution where all data points are positioned in a single cluster. Additionally, use of this reference distribution for data with no noise would allow to measure the number of clusters.

Suppose that the dataset could be approximated as consisting of N_c, where N_c is the number of cluster in the dataset, well separated delta functions, each once centered at position c_j, such that the distance between any pair of clusters is $> 2r$ (i.e. larger than twice our current Parzen window bandwidth). We shall call this distribution $\tilde{q}(x)$, and define it as:

$$\tilde{q}(x) = \frac{1}{N_c} \sum_{j=1}^{N_c} \delta(x - c_j) \;\; s.t. \;\; | \, c_i - c_j \, | > 2r \;\; \forall i \neq j. \tag{9}$$

To generate $q(x)$, we convolve this distribution with the Parzen window function,

$$q(x) = \tilde{q}(x) * \frac{I(| \, x \, | < r)}{\pi r^2} = \frac{1}{N_c} \sum_{j=1}^{N_c} \frac{I(| \, x - c_j \, | < r)}{\pi r^2}, \tag{10}$$

where we have made use of the sifting property of delta functions. It is desired to evaluate this for each observed localisation x_i, so as to be able to estimate the Rényi divergence. To perform this calculation exactly, we would need to explicitly set the position of all cluster centres c_j, or take a more Bayesian approach by defining priors and marginalising them out. Either way, this exact calculation will require an expensive numerical optimisation or integration.

Instead, we approximate this calculation by explicitly assuming that each observed localisation corresponds to one (and only one) cluster. Combining this with the assumption that the clusters are separated by at least a distance of $2r$, this indicates there is exactly one cluster center c_j which is within a distance r of x_i. This gives an immediate closed form evaluation for $q(x_i)$:

$$q(x_i) = \frac{1}{N_c} \sum_{j=1}^{N_c} \frac{I(| \, x_i - c_j \, | < r)}{\pi r^2} = \frac{1}{N_c} \left(0 + 0 + \ldots 0 + \frac{1}{\pi r^2} + 0 + \ldots + 0 \right) = \frac{1}{N_c \pi r^2}. \tag{11}$$

An immediate criticism of this choice of approximation is that it is improper – it cannot be normalised such that $\int_{x_i \in \mathbb{R}^2} q(x_i) dx_i = 1$. This is true – however, we point out that we are *explicitly* conditioning this calculation on the knowledge that it is likely that each x_i we are called to evaluate *in practise* is a localisation generated by either a cluster-like object, or background noise. Either way, they are confined to a finite region (the extent of the image).

Moreover, as this approximation of the value of $q(x_i)$ shares a very similar form to the Parzen window estimator of $p(x)$, their ratio possesses some nice properties, as we shall now show.

The Rényi divergence has a general equation for $\alpha \neq 1 \wedge \alpha > 0$ and a special case for $\alpha = 1$. For the purpose of cluster analysis we used only the general case, which can be written using equation (1) by substituting the $p(x_i)$ and $q(x_i)$ in with formulas given by equations (8) and (11):

$$D_\alpha = \frac{1}{\alpha - 1} \ln \left[\frac{1}{N} \sum_{i=1}^{N} \left(\frac{\frac{1}{N} \sum_{j=1}^{N} \frac{1}{\pi r^2} I(d_{ij} < r)}{\frac{1}{\pi r^2 N_c}} \right)^{\alpha - 1} \right], \tag{12}$$

which can be rewritten by splitting the exponentiation base and moving the constant term N (for the number of points in the data) outside of the sum over i and j:

$$D_\alpha - \frac{1}{\alpha - 1} \ln \left({N_c}^{\alpha - 1} \right) = \frac{1}{\alpha - 1} \ln \left[\frac{\sum_{i=1}^{N} \left(\sum_{j=1}^{N} I(d_{ij} < r) \right)^{\alpha - 1}}{N^\alpha} \right]. \tag{13}$$

Equation (13) can be further simplified using a property of logarithms to change division into the difference of two logarithms:

$$D_\alpha - \frac{1}{\alpha - 1} \ln \left({N_c}^{\alpha - 1} \right) = \frac{1}{\alpha - 1} \left[\ln \left(\sum_{i=1}^{N} \left(\sum_{j=1}^{N} I(d_{ij} < r) \right)^{\alpha - 1} \right) - \alpha \ln (N) \right]. \tag{14}$$

The equation (14) is used in the calculations. Use of this equation helps to maintain optimal memory allocation for the Rényi divergence calculation. This is achieved by splitting the equation into separate parts which are responsible for counting the points inside a radius and storing the normalising term depending on the number of points in the dataset.

4 Supplementary Note: The Rényi divergence for a uniform reference distribution

The Rényi divergence using a sampling approximation, is given by equation (7). For a data distribution given by equation (8) and a uniform reference distribution, indexed by U for a uniform distribution:

$$q^{\mathrm{U}}(x_i) = \frac{1}{A}, \tag{15}$$

where A is the image area. A uniform reference distribution $q^U(x_i)$ is the same reference distribution used by Ripley's K function. The Rényi divergence is equal to:

$$D_\alpha^{\mathrm{U}} = \frac{1}{\alpha-1} \ln \left[\frac{1}{N} \sum_{i=1}^{N} \left(\frac{\frac{1}{N}\sum_{j=1}^{N} \frac{1}{\pi r^2} I(d_{ij} < r)}{\frac{1}{A}} \right)^{\alpha-1} \right]. \tag{16}$$

Both the uniform and, previously considered, clustered reference distributions are point independent (their value does not depend for the particular point in the image they are calculated for). A uniform distribution is constant for the whole image and a clustered distribution depends on the number of cluster in the whole image. Thus, one can separate equation (16) into point-specific and point-independent parts. This means that all of the parameters which have no dependency on i and/or j can be excluded from the summation. The number of points N is constant:

$$D_\alpha^{\mathrm{U}} = \frac{1}{\alpha-1} \ln \left[\frac{1}{N} \left(\frac{1}{N}\right)^{\alpha-1} \sum_{i=1}^{N} \left(\frac{\sum_{j=1}^{N} \frac{1}{\pi r^2} I(d_{ij} < r)}{\frac{1}{A}} \right)^{\alpha-1} \right]. \tag{17}$$

The area A of the image is also constant

$$D_\alpha^{\mathrm{U}} = \frac{1}{\alpha-1} \ln \left[\frac{1}{N} \left(\frac{1}{N}\right)^{\alpha-1} A^{\alpha-1} \sum_{i=1}^{N} \left(\sum_{j=1}^{N} \frac{1}{\pi r^2} I(d_{ij} < r) \right)^{\alpha-1} \right], \tag{18}$$

and the area withing which points are counted πr^2 is also independent from i and j:

$$D_\alpha^{\mathrm{U}} = \frac{1}{\alpha-1} \ln \left[\frac{1}{N} \left(\frac{1}{N}\right)^{\alpha-1} A^{\alpha-1} \left(\frac{1}{\pi r^2}\right)^{\alpha-1} \sum_{i=1}^{N} \left(\sum_{j=1}^{N} I(d_{ij} < r) \right)^{\alpha-1} \right]. \tag{19}$$

Equation (19) can be written as:

$$\begin{aligned} D_\alpha^{\mathrm{U}} &= \frac{1}{\alpha-1} \left\{ \ln \left[\left(\frac{A}{\pi r^2}\right)^{\alpha-1} \right] + \ln \left[\left(\frac{1}{N}\right)^{\alpha} \sum_{i=1}^{N} \left(\sum_{j=1}^{N} I(d_{ij} < r) \right)^{\alpha-1} \right] \right\} \\ &= \frac{1}{\alpha-1} \ln \left[\left(\frac{A}{\pi r^2}\right)^{\alpha-1} \right] + \frac{1}{\alpha-1} \ln \left[\left(\frac{1}{N}\right)^{\alpha} \sum_{i=1}^{N} \left(\sum_{j=1}^{N} I(d_{ij} < r) \right)^{\alpha-1} \right]. \end{aligned} \tag{20}$$

The Rényi divergence (for $\alpha \in [0, \infty) \wedge \alpha \neq 1$), calculated for a clustered reference distribution using equation (**??**) (as discussed in Supplementary Note 3), can also be arranged into data constant and clustering counting parts:

$$D_\alpha = \frac{1}{\alpha-1} \ln \left({N_c}^{\alpha-1} \right) + \frac{1}{\alpha-1} \ln \left[\left(\frac{1}{N}\right)^{\alpha} \sum_{i=1}^{N} \left(\sum_{j=1}^{N} I(d_{ij} < r) \right)^{\alpha-1} \right], \tag{21}$$

where N is the number of all points in the data set, $I(d_{ij} < r)$ is an indicator function, πr^2 is the area of a cluster, and N_c is the number of clusters. The term $\frac{1}{\alpha-1} \ln \left({N_c}^{\alpha-1} \right)$ is constant with respect to the points on the image and provides an offset to the divergence function. The Rényi divergence defined for a uniform distribution differs only by a constant value $\frac{1}{\alpha-1} \ln \left[\left(\frac{A}{\pi r^2}\right)^{\alpha-1} \right]$ from the Rényi divergence calculated for clustered distribution. Thus, the Rényi divergence for a uniform reference distribution can be written as proportional to:

$$D_\alpha^{\mathrm{U}} \propto \frac{1}{\alpha-1} \ln \left[\left(\frac{1}{N}\right)^{\alpha} \sum_{i=1}^{N} \left(\sum_{j=1}^{N} I(d_{ij} < r) \right)^{\alpha-1} \right], \tag{22}$$

This means that the Rényi divergence calculated for the uniform distribution with equation (22) can provide the same results for clustering analysis as the Rényi divergence calculated for a clustered distribution.

Ripley's K function can be derived from the Rényi divergence calculated for a uniform distribution and $\alpha = 2$:

$$D_2 = \frac{1}{2-1} \ln \left[\frac{1}{N} \sum_{i=1}^{N} \left(\frac{\frac{1}{N} \sum_{j=1}^{N} \frac{I(d_{ij}<r)}{\pi r^2}}{\frac{1}{A}} \right)^{2-1} \right], \tag{23}$$

now the $\frac{1}{N}$ from before the summation can be moved inside it giving:

$$D_2 = \ln \left[\sum_{i=1}^{N} \left(\frac{\frac{1}{N} \sum_{j=1}^{N} \frac{I(d_{ij}<r)}{\pi r^2}}{\frac{N}{A}} \right) \right]. \tag{24}$$

Now, writing $\frac{N}{A}$ as λ we get:

$$D_2 = \ln \left[\sum_{i=1}^{N} \left(\frac{\frac{1}{N} \sum_{j=1}^{N} \frac{I(d_{ij}<r)}{\pi r^2}}{\lambda} \right) \right]. \tag{25}$$

By removing the logarithm this can be written as:

$$e^{D_2} = \sum_{i=1}^{N} \left(\frac{\frac{1}{N} \sum_{j=1}^{N} \frac{I(d_{ij}<r)}{\pi r^2}}{\lambda} \right), \tag{26}$$

and multiplying both sides of the equation by πr^2 gives:

$$e^{D_2} \pi r^2 = \lambda^{-1} \left[\sum_{i=1}^{N} \sum_{j=1}^{N} I(d_{ij} < r) \right] / N, \tag{27}$$

which is equal to Ripley's K function. Ripley's K function is a special case of the Rényi divergence.

5 Supplementary Note: DNA origami size measurements with the Rényi divergence and Ripley's H function

The DNA origami structures had been designed to have a specific shape and size (30), here we used rectangular plates with dimensions of 60×90 nm. However, both the Rényi divergence and Ripley's H function use a circle to measure the local clustering patterns, which means that the cluster size measured by these methods will be the radius of a circle with a relation to the size of the structure itself but it will not be its actual size. The potential values of the measured radius can be estimated: the measured cluster should not be smaller than a circle fitting inside the rectangle or bigger than the circumcircle (see Supplementary Fig. 4). The lower limit, or minimal expected radius, underestimates the cluster size, as a big part of the structure with a high density of molecules is not included in a potential cluster. The minimal radius was given as half of the length of the shorter side of the rectangle. The higher limit and maximal expected radius for the measured cluster radius was a circumcircle (see Supplementary Fig. 4a) with radius given as half of the diagonal of the rectangle: $r = \frac{\sqrt{x^2+y^2}}{2}$. For the 60×90 nm plates the minimal expected radius was equal to 30 nm and the maximal expected radius was equal to 54 nm. We have also simulated DNA origami plates measuring 16x24 pixels with a pixel size of 3.75 nm (this preserved the size and shape of real 60×90 nm plates, each dataset had 10 clusters and S/N 29) to account for the difference between the measured cluster radius and the maximal expected cluster radius.

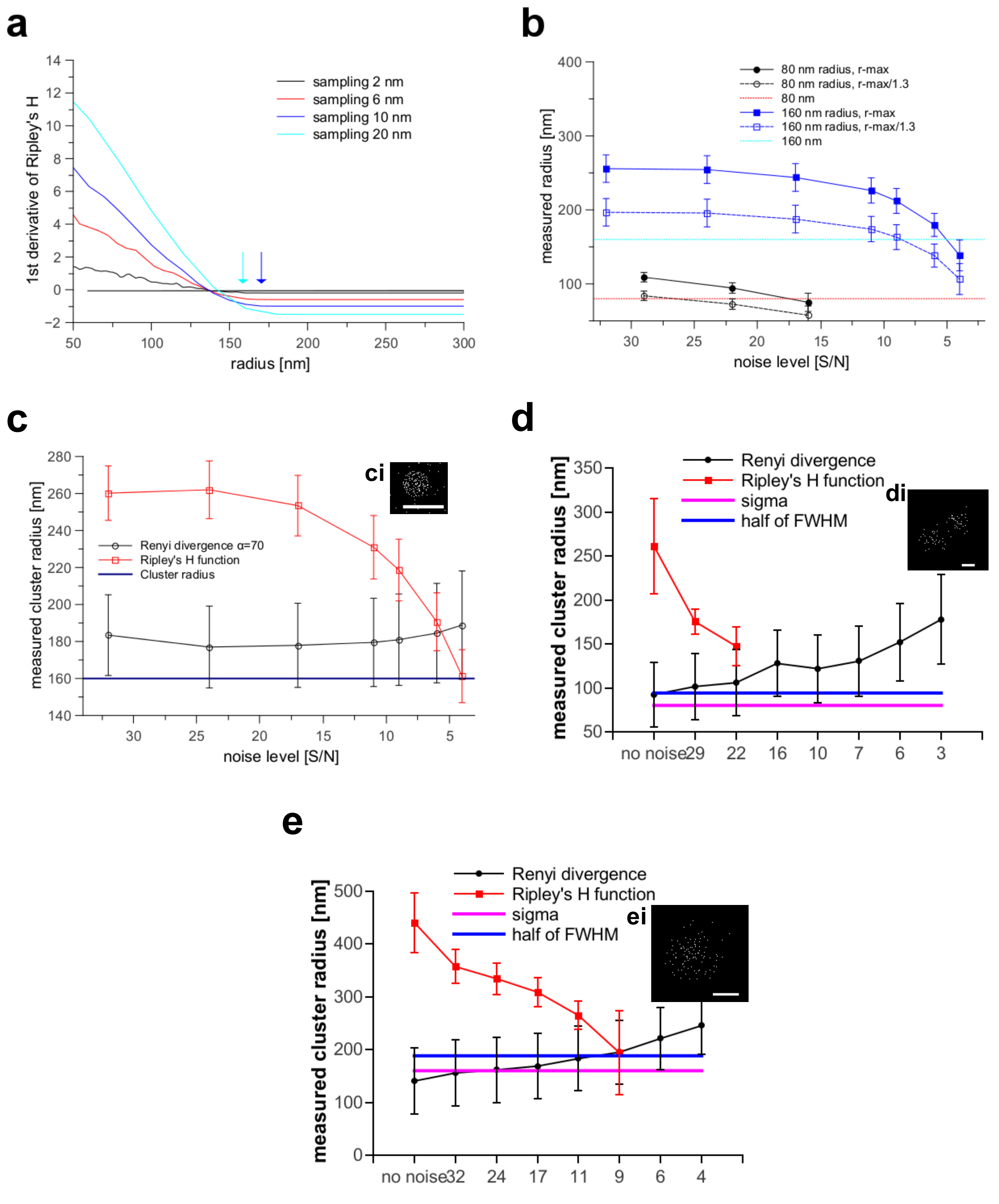

Supplementary Figure 1. Results of cluster radius measurements for simulated data and problem with bias removal from the cluster radius measurements for RipleyâĽ™s H function. a) Method measuring cluster size as the half of the radius for which the first derivative of Ripley's H function is equal to -1 (marked with arrows). The first derivative is calculated numerically, thus for different sampling ratios the first derivative may appear in different positions (marked with different colour arrows). b) Removing bias from the radius measurements using a coefficient. The radius values measured are much closer to the actual cluster radius for datasets with high S/N. However, the results acquired at lower S/N fail more quickly than standard method of radius measurement by detecting the maximum of Ripley's H function. Cluster radius measurement of simulated circular clusters: c) clusters with 160 nm radius and uniform point distribution through the cluster and clusters with Gaussian point distribution sigma d) 80 nm and e) 160 nm. For each noise the same number of datasets was used (100,000 for uniform and 50,000 for Gaussian distributed clusters). The error bars are the standard deviations. The inlets ci), di), and ei) are examples of the simulated clusters. Blue lines mark the cluster radius. Scale bar: ci) 500 nm, di) 160 nm, and ei) 320 nm.

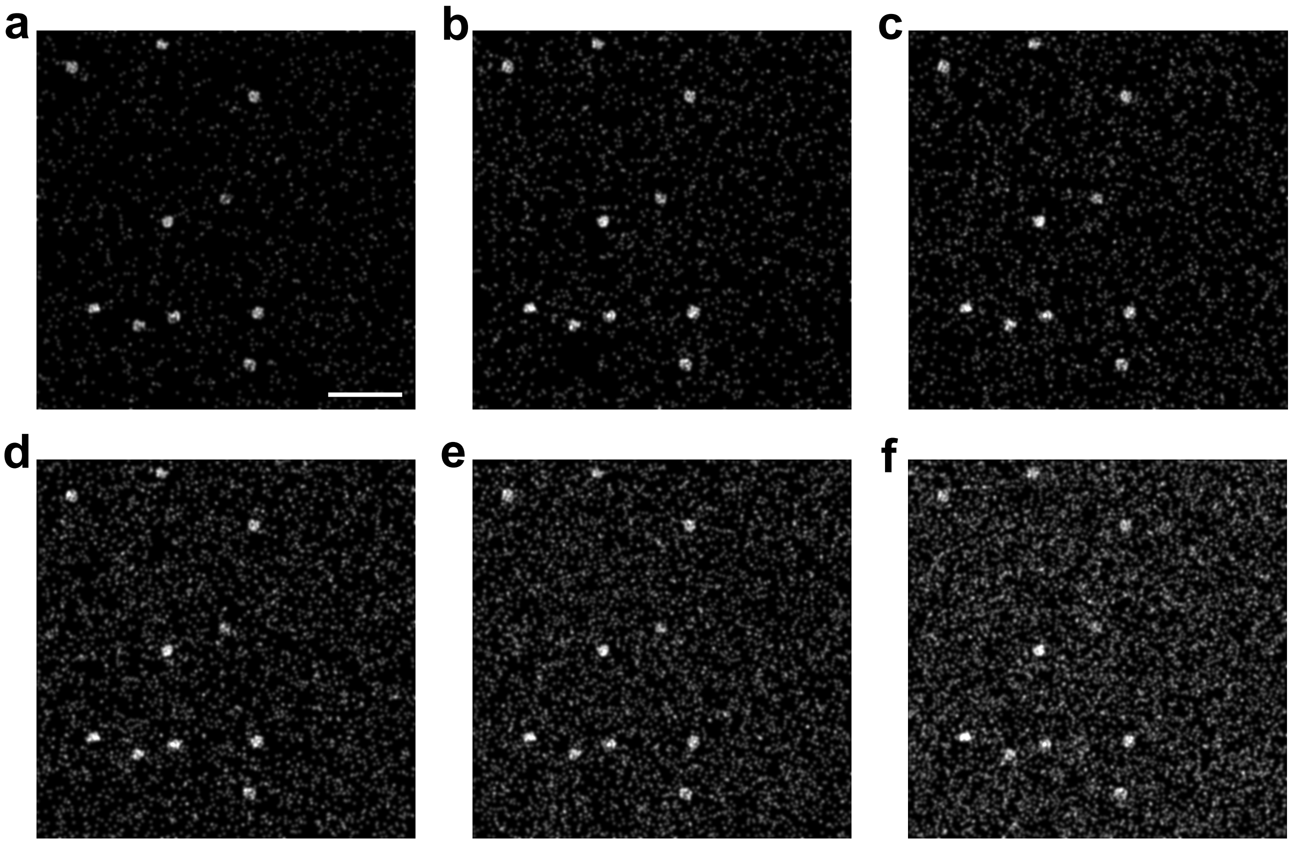

Supplementary Figure 2. Examples of simulated data sets with 10 clusters (radius 80 nm). Noise was added to each data set, in the form of false/background localisations: a) 0.5% of pixels are noise, which corresponds to a signal-to-noise (S/N) of 29, b) 0.74% – S/N 22, c) 1.0% – S/N 16, d) 1.5% – S/N 10, e) 2.0% – S/N 7, and f) 3.0% – S/N 6. For improved visibility (in this figure only) the images were blurred (with Gaussian blur, sigma 20 nm). Scale bar 1 μm.

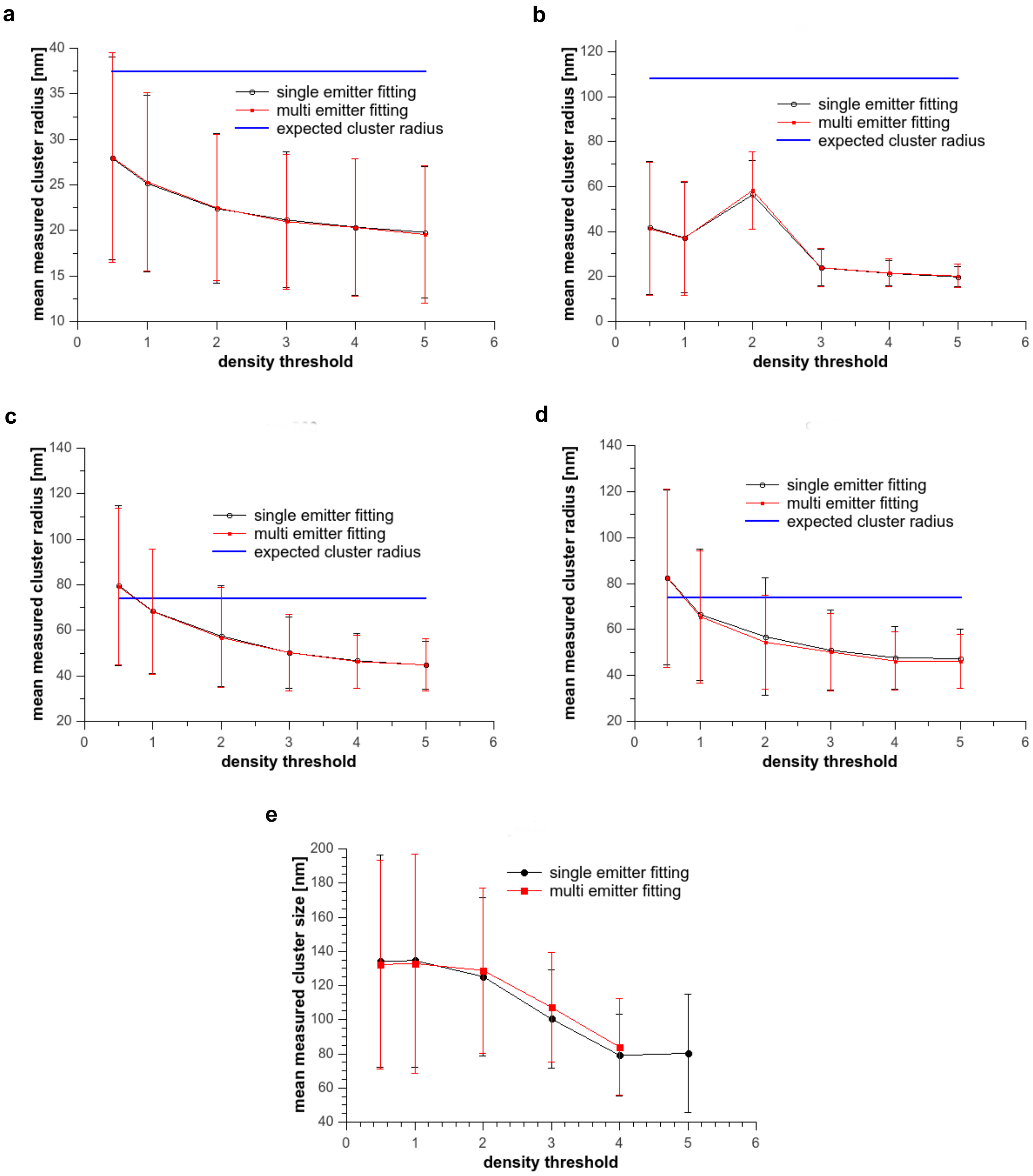

Supplementary Figure 3. The mean cluster radius measured measured for different density threshold parameters with SR-Tesseler. Cluster radius measured for a) DNA origami, b) vaccinia virus particles, c and d) clathrin coated pits with 1:200 and 1:500 anti-clathrin antibody density, e) podosomes for single- and multi-emitter fitting. The error bars are the standard deviations.

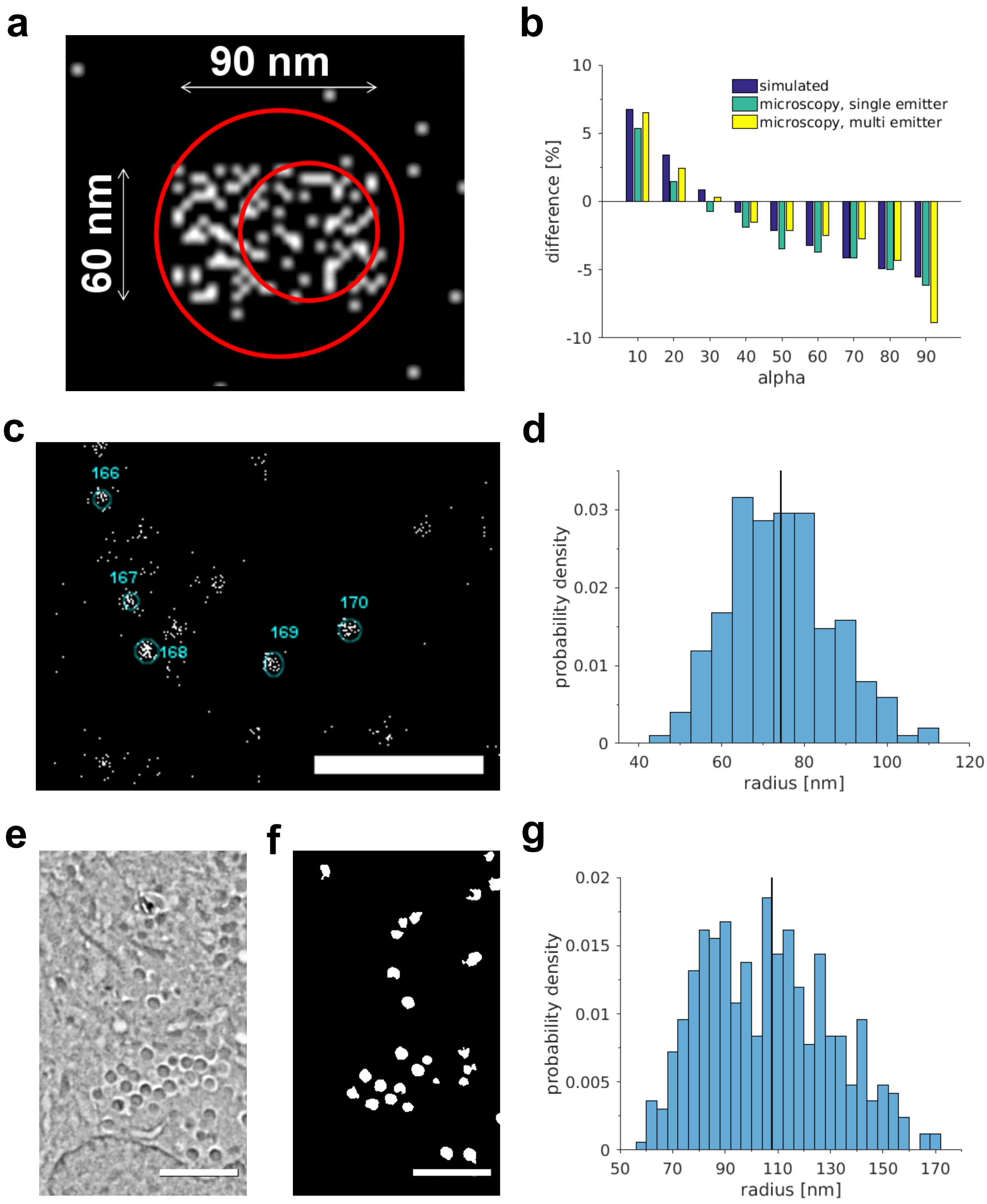

Supplementary Figure 4. Cluster radius measurement validation for DNA origami, clathrin-coated pits, and vaccinia virus particles. a) Simulated DNA plate and cluster radius estimation. The radius of the circle passing through the corners of the DNA origami plate can be calculated as half of the diagonal, and the inner-circle radius is equal to half of the length of the shorter side of the plate. b) The percentage difference between the maximal expected radius and the mean radius measured with the Rényi divergence for simulated and experimental microscopy data. Especially for higher values of α the values measured for simulated and experimental data are very similar. c) Visual assessment of the size of clusters in localisation microscopy image of clathrin coated pits (single-emitter fitting and 1:200 anti-clathrin antibody concentration), a fragment of the analysed image. Each cluster was numbered and their radius estimated (marked with a circle). Scale bar 1 μm. d) Results of the visual radius estimation. The mean value, 74±13 nm, is marked with black line. Vaccinia virus particles e) a region of an electron microscopy image f) particle identifications, g) radius measured for the identified particles, the mean value, 108±29 nm, is marked with a black line. Scale bar 1 μm.

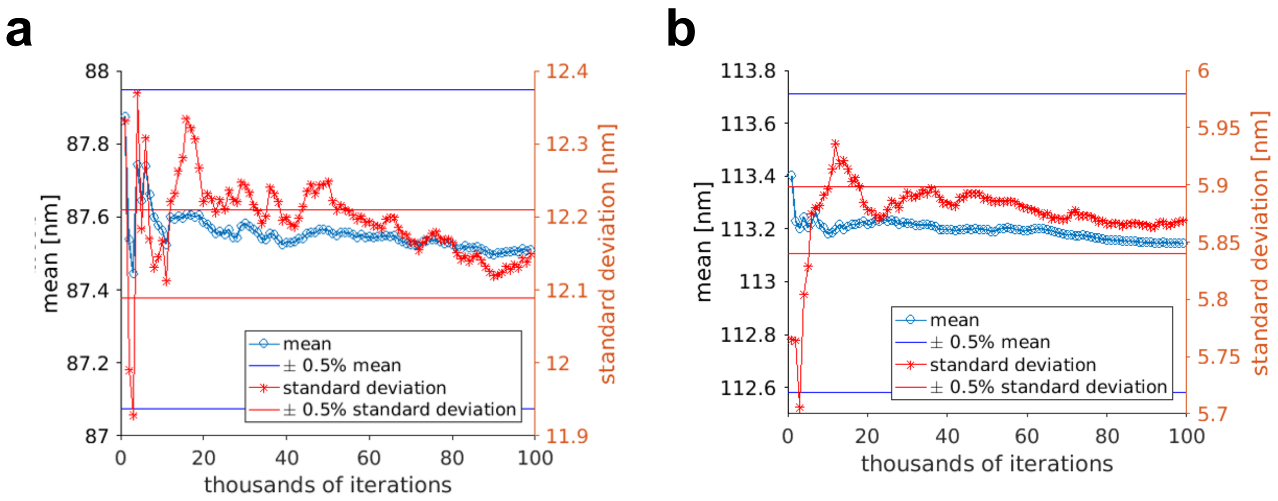

Supplementary Figure 5. An example of statistical analysis of simulated cluster datasets. Here the analysis was performed for datasets with 10 clusters with 80 nm radius and S/N 29. The measured values of mean radius (blue) and the standard deviation (red) measured for different number of Monte Carlo repetitions, for a) the Rényi divergence and b) Ripley's H function. The horizontal lines, blue and red, mark the 0.5% change in the values of mean and the standard deviation respectively.

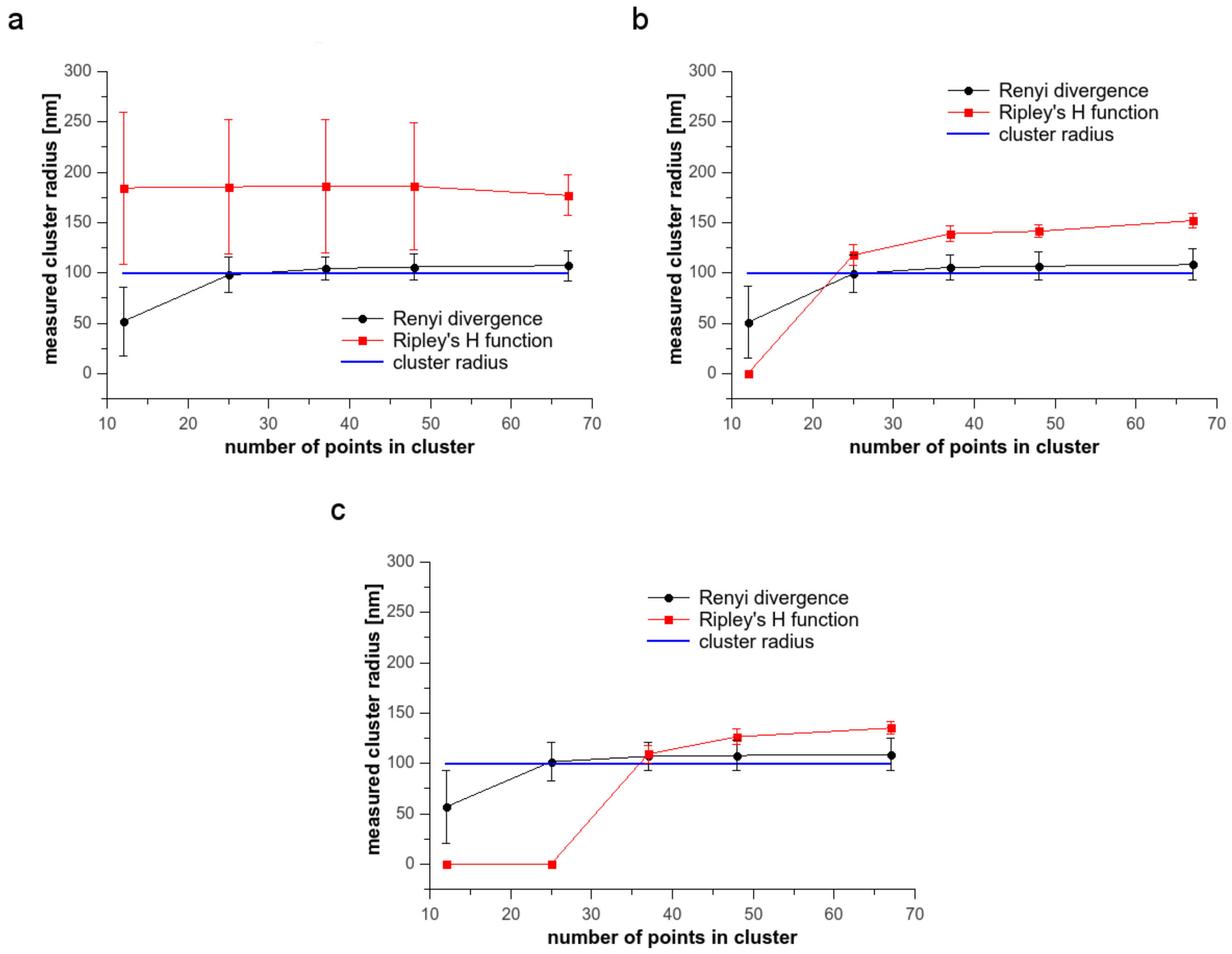

Supplementary Figure 6. Simulated clustered datasets with 10 clusters with 100 nm radius and different number of points in the cluster. The simulated clusters had from 12 to 67 points. These datasets were simulated a) without and b-c) with noise. For b and c) we have simulated noise at constant level of around 1300 and 2600 points per dataset (this corresponds to S/N 29 and S/N 22 for clusters with 35 points). The Rényi divergence provided measurement closer to the actual cluster radius than Ripley's H function for all investigated densities of points in the cluster. The error bars are the standard deviations.

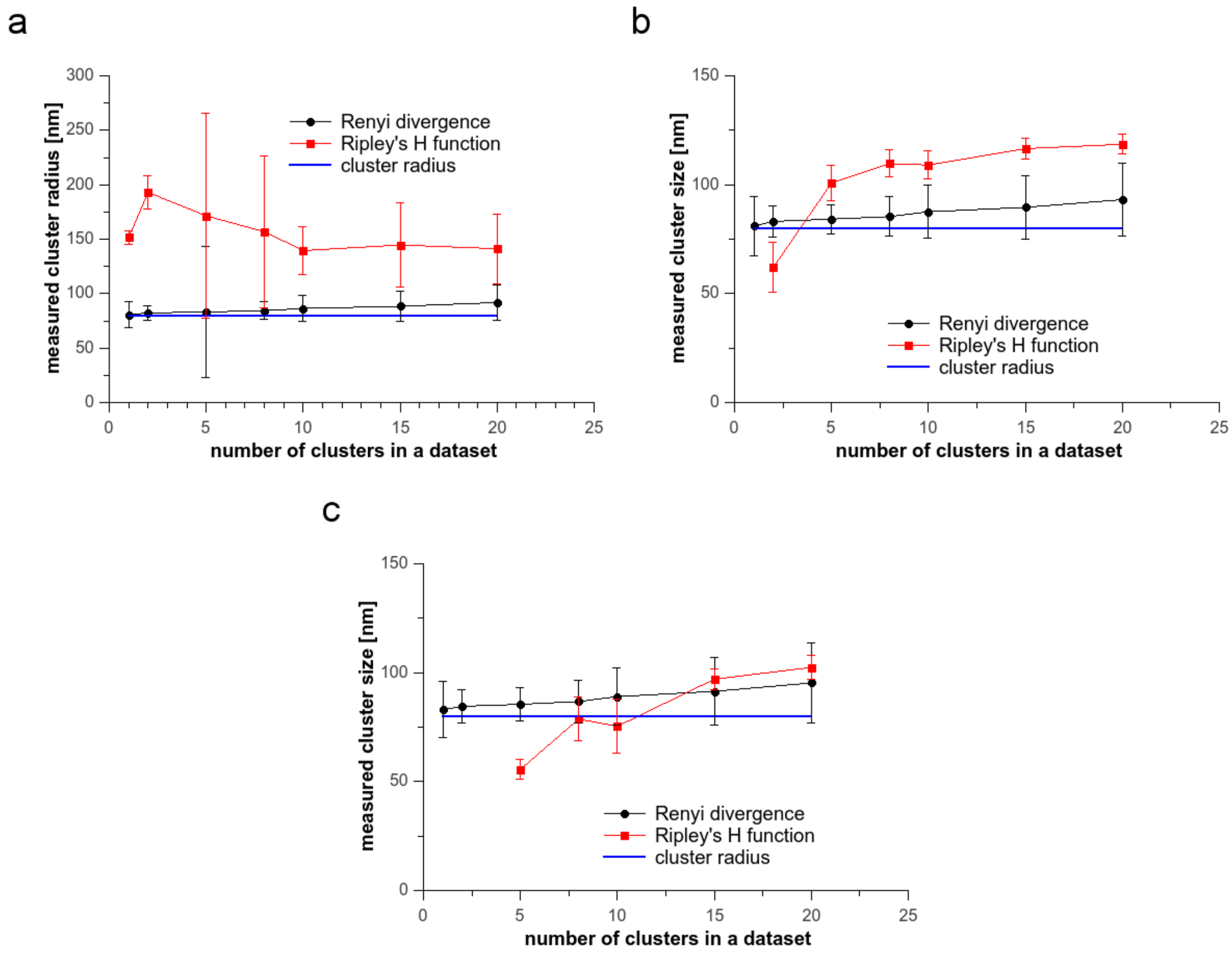

Supplementary Figure 7. Simulated clustered datasets containing from 1 to 20 clusters with 80 nm radius for three different noise levels. Simulations with a) no noise, b) S/N 29, and c) S/N 16. The Rényi divergence provided more accurate and stable measurements of cluster size than Ripley's H function. It should be also noted that Ripley's H function struggles with data without noise and is unable to provide cluster radius measurement for datasets with small number of clusters with noise. The error bars are the standard deviations.

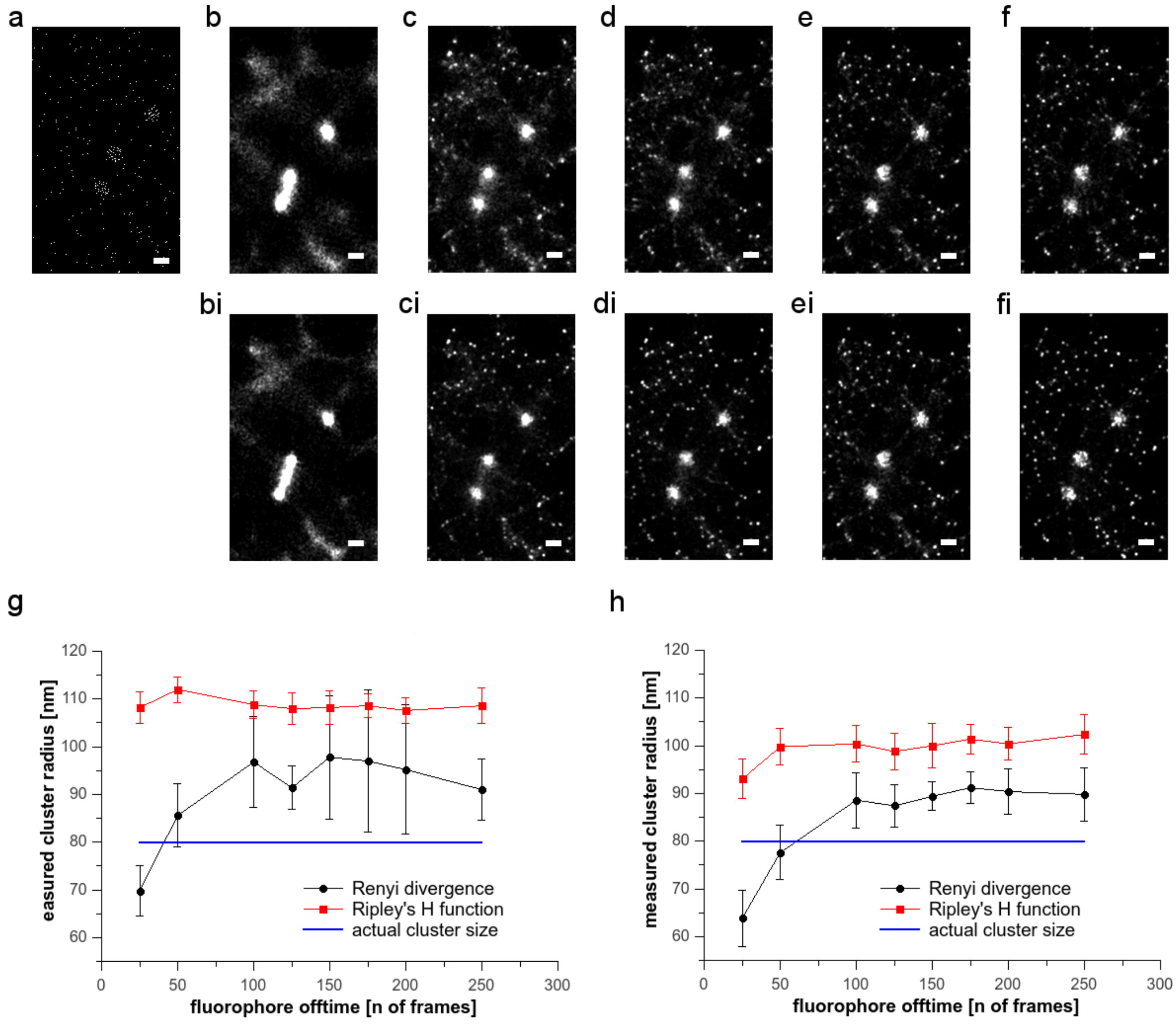

Supplementary Figure 8. Simulated single molecule images and cluster radius measurement. a) A fragment of an image with 10 clusters with 80 nm radius used to define fluorophore positions for single molecule image simulation. b-fi) Images reconstructed for different density of simulated single molecules (achieved by changing the amount of frames each molecule spends in the dark state) and using ThunderSTORM with single- (top row) and multi-emitter (bottom row, indexed "i"). Examples of the images reconstructed for different molecule offtime: b and bi) 25 frames, c and ci) 100 frames, d and di) 150 frames, e and ei) 200 frames, f and fi) 250 frames. Few artefacts, especially for high density single molecule data can be observed (i.e. merging of clusters in close proximity), and uneven noise background. Scale bar: 160 nm. The mean measured cluster radius for simulated single molecules with different offtime localised using g) single- and h) multi-emitter fitting in ThunderSTORM. We have observed similar behaviour for the Rényi divergence and Ripley's H function for both of fitting methods. The error bars are the standard deviations.

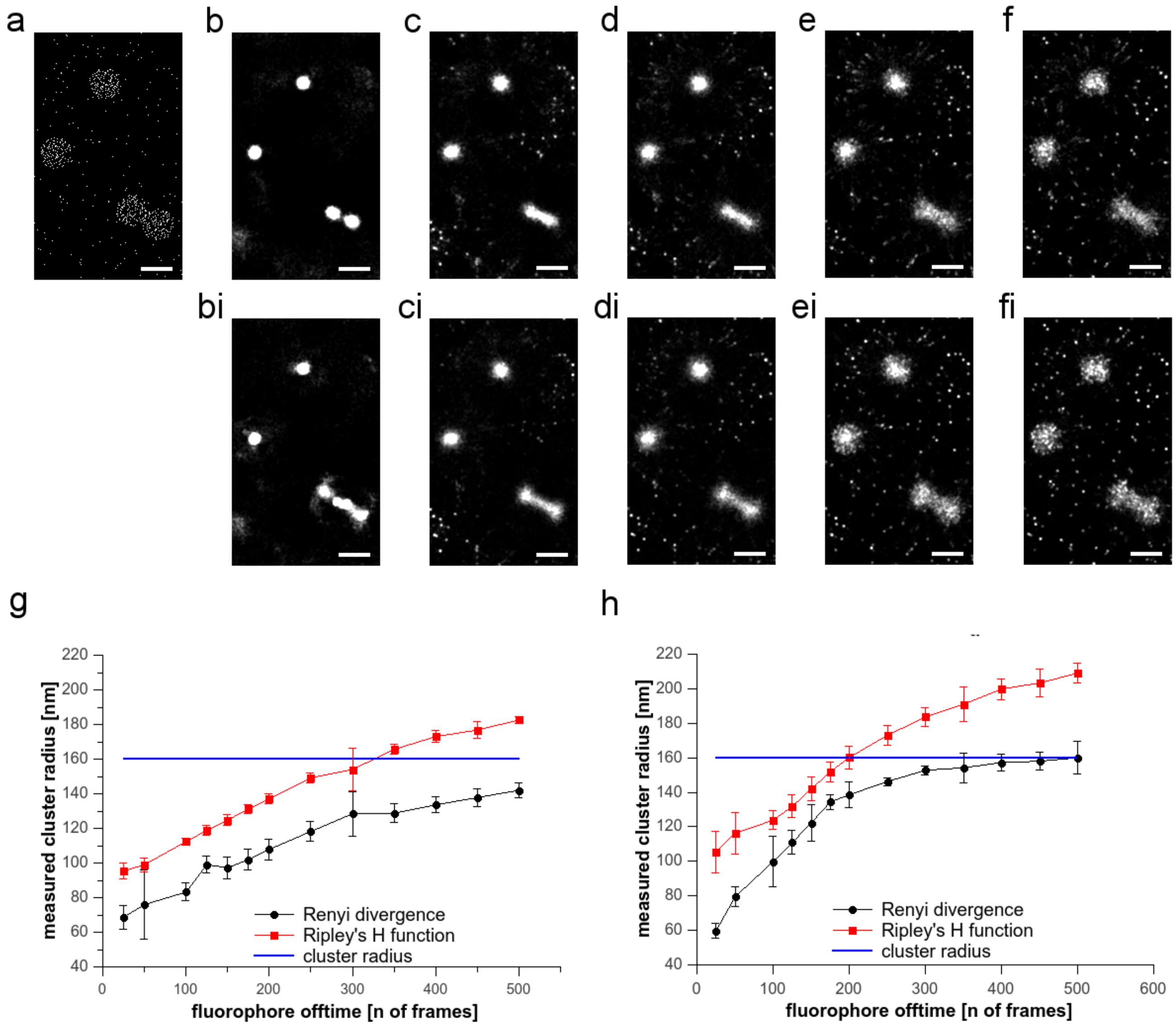

Supplementary Figure 9. Simulated single molecule images with different molecule density and cluster radius measurement. a) A fragment of an image with 10 clusters with 160 nm radius used to define fluorophore positions for single molecule image simulation. b-fi) Images reconstructed for different density of simulated single molecules (achieved by changing the amount of frames each molecule spends in the dark state) and using ThunderSTORM with single- (top row) and multi-emitter (bottom row, indexed "i"). Examples of the images reconstructed for different molecule offtime: b and bi) 25 frames, c and ci) 125 frames, d and di) 200 frames, e and ei) 350 frames, f and fi) 500 frames. For higher density we observed artefacts in the rendering of clusters (cluster merging, sharpening, halo formation) and noise (formation of mesh structure in the background). Scale bar: 320 nm. The mean measured cluster radius for simulated single molecules with different offtime localised using ThunderSTORM with g) single- and h) multi-emitter fitting. For lower density data the size of the reconstructed structure closely matches the actual structure size a discrepancy in size can be observed for higher density data. The error bars are the standard deviations.

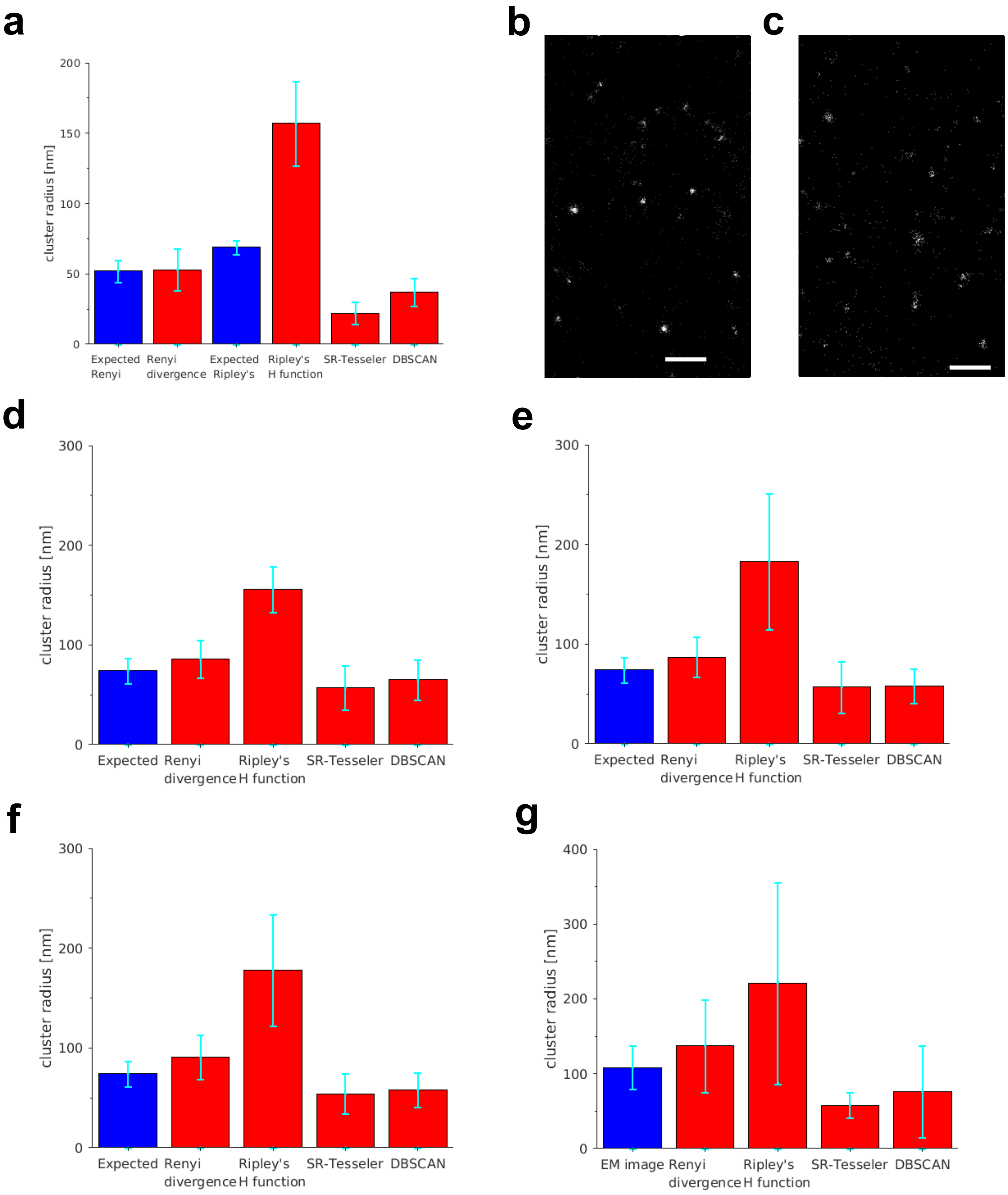

Supplementary Figure 10. Comparative results of cluster radius measurement for DNA-origami, clathrin coated pits, and vaccinia virus particles. The average radius was measured with the Rényi divergence, Ripley's H function, SR-Tesseler, and DBSCAN. a) Expected and average measured cluster radius for five DNA-origami samples reconstructed with multi-emitter fitting. The average cluster radii measured for experimental data are 53±15 nm (the Rényi divergence), 157±30 nm (Ripley's H function), 22±8 nm (SR-Tesseler), and 37±10 nm (DBSCAN). Single-emitter fitting reconstructed images of clathrin coated pits stained with b) 1:200 and c) 1:500 anti-clathrin antibody concentration. A slightly higher signal level was observed for samples prepared with 1:200 antibody concentration. Scale bar 1 μm. d-f) Average cluster size for multi-emitter fitting for d) 1:200 antibody-concentration, 86±19 nm, 156±58 nm, 57±22 nm, and 65±20 nm, e) single-emitter fitting 1:500 antibody concentration, 91±20 nm, 213±68 nm, 57±26 nm, 58±17 nm, and f) multi-emitter fitting for 1:500 antibody concentration for three clathrin coated pit samples, 91±22 nm, 178±56 nm, 54±20 nm, and 58±17 nm for the Rényi divergence, Ripley's H function, SR-Tesseler and DBSCAN respectively. g) The average radius for four vaccinia virus particles samples, here localisation results were analysed with multi-emitter fitting giving 137±62 nm, 221±135 nm, 58±17 nm, and 76±61 nm respectively. Error bars are the standard deviation.

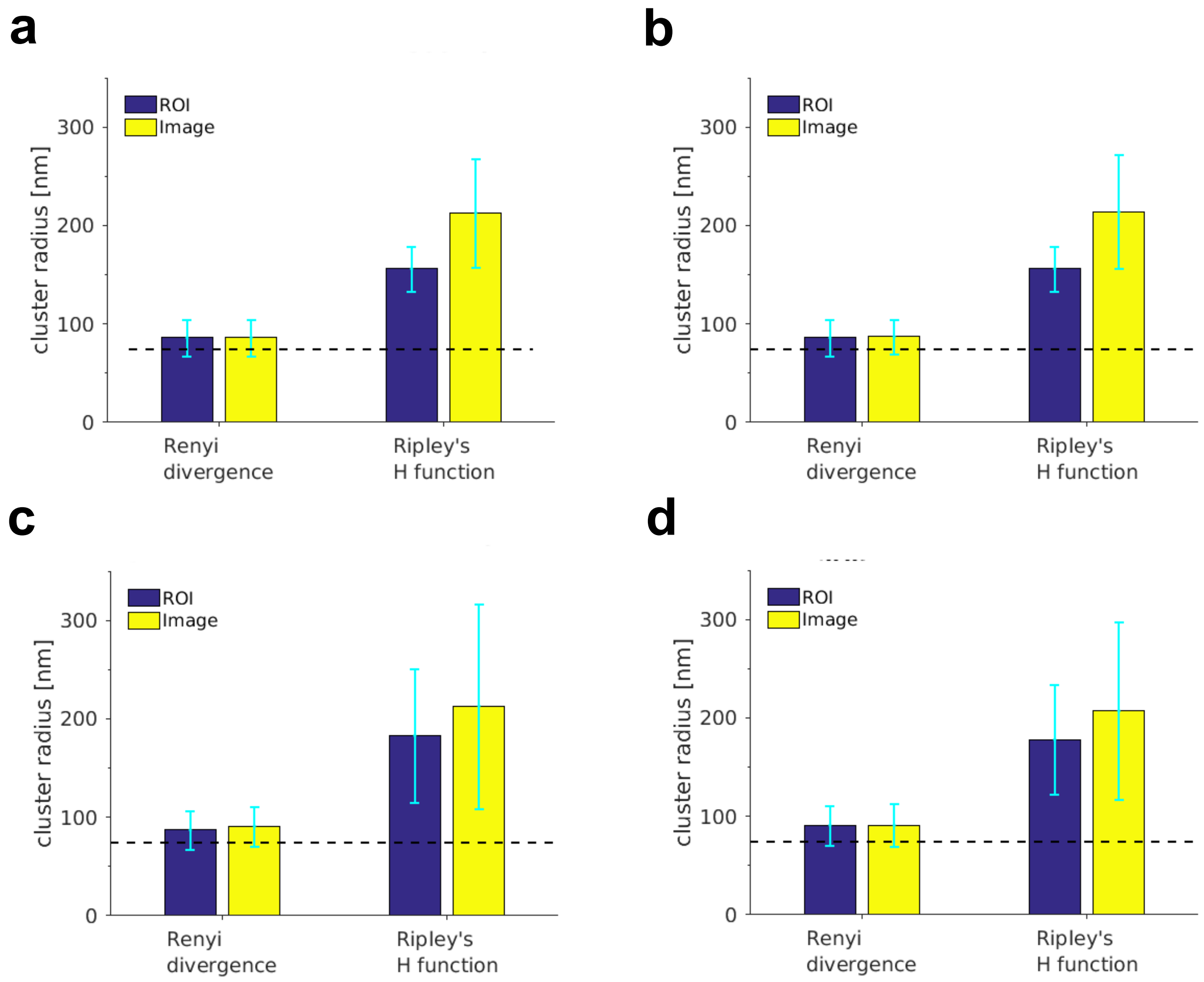

Supplementary Figure 11. Region of interest selection for the Rényi divergence and Ripley's H function for analysis of clathrin coated pit data. The cluster analysis of localisation microscopy datasets was performed for a selected region of interest (ROI). For clathrin coated pits the ROIs were either selected to include the cell, or used the whole dataset (Image). The mean cluster radius measured with Ripley's H function changed depending on the selected region of interest, while the mean radius measured with the Rényi divergence was either changed by a very small amount or unchanged. Clathrin coated pit samples were prepared with 1:200 anti-clathrin antibody concentration. Molecule localisation was performed with a) single-emitter and b) multi-emitter fitting, and samples prepared with 1:500 concentration with c) single-emitter and d) multi-emitter fitting. The dash line indicates the expected radius value of 74$\pm$13 nm.

Table 1. The p-values of the two-sample Kolmogorov-Smirnov test (significance level 5%) of the cluster radius values measured using the Rényi divergence, Ripley's H function, SR-Tesseler, DBSCAN and radius estimation methods. The cluster radius measured with these clustering analysis methods for localisation microscopy data (LM) was compared with the results of simulations (for DNA origami), visual radius assessment (visual) for clathrin coated pits, and the radius measured for electron microscopy image (EM).

DNA origami

Type of emitter fitting	Rényi simulated and LM	Ripley's H f. simulated and LM	Rényi and Ripley's H f. LM	Rényi and SR-Tesseler LM	Rényi and DBSCAN LM
single	8.78×10^{-8}	7.35×10^{-54}	6.30×10^{-27}	6.84×10^{-37}	6.22×10^{-12}
multi	6.30×10^{-6}	7.07×10^{-54}	7.82×10^{-28}	9.21×10^{-39}	4.14×10^{-13}

Clathrin coated pits, 1:200 antibody concentration

Type of emitter fitting	Visual and Rényi LM	Visual and Ripley's H f. LM	Visual and SR-Tesseler LM	Visual and DBSCAN LM	Rényi LM and Ripley's H f. LM	Rényi LM and SR-Tesseler LM	Rényi LM and DBSCAN LM
single	2.65×10^{-4}	2.40×10^{-28}	1.18×10^{-65}	1.47×10^{-26}	2.61×10^{-18}	1.80×10^{-19}	1.73×10^{-9}
multi	1.09×10^{-4}	1.29×10^{-29}	1.26×10^{-66}	2.68×10^{-27}	1.94×10^{-18}	1.74×10^{-18}	6.65×10^{-11}
**Clathrin coated pits, 1:500 antibody concentration**							
single	0.0024	4.49×10^{-25}	2.66×10^{-60}	8.09×10^{-51}	5.33×10^{-14}	1.41×10^{-13}	9.64×10^{-12}
multi	3.22×10^{-5}	4.49×10^{-25}	6.08×10^{-63}	5.48×10^{-52}	3.00×10^{-12}	1.25×10^{-14}	7.38×10^{-12}

Vaccinia virus particles

Type of emitter fitting	EM and Rényi LM	EM and Ripley's H f. LM	EM and SR-Tesseler LM	EM and DBSCAN LM	Rényi LM and Ripley's H f. LM	Rényi LM and SR-Tesseler LM	Rényi LM and DBSCAN LM
single	0.035	5.36×10^{-4}	1.62×10^{-51}	3.08×10^{-61}	0.088	2.09×10^{-5}	0.0075
multi	0.014	0.0018	8.33×10^{-50}	8.79×10^{-61}	0.40	2.12×10^{-5}	0.0020

Podosome cores

Type of emitter fitting	Rényi LM and Ripley's H f. LM	Rényi LM and SR-Tesseler LM	Rényi LM and DBSCAN LM
single	7.16×10^{-7}	1.55×10^{-8}	1.58×10^{-12}
multi	1.32×10^{-6}	2.61×10^{-7}	2.01×10^{-15}

Table 2. Cluster radius measured with the Rényi divergence, Ripley's H function, SR-Tesseler, and DBSCAN for localisation microscopy data. For each sample an expected radius of the structure is given. For DNA origami we simulated DNA origami plates and measured the radius with the Rényi divergence (*) and Ripley's H function (**). The radius measured with the Rényi divergence is very close to the radius of a circumcircle (54 nm), see Supplementary Note 5. For clathrin coated pits the radius was estimated using electron microscopy images presented in (38) (†) and via visual inspection of localisation microscopy image (‡). The clathrin coated pits radius is lowered for the visual inspection due to existence of false clusters in the background. For vaccinia virus particles, the radius was measured using electron microscopy images. The number of analysed datasets is indicated in the last column. However, it should be noted that for analysis of podosome core data Ripley's H function only provided a cluster radius measurement for 17 datasets analysed with single emitter fitting and 13 datasets for multi-emitter fitting.

sample type	Expected radius [nm]	Type of emitter fitting	The Rényi divergence [nm]	Ripley's H function [nm]	SR-Tesseler	DBSCAN	N. of data-sets
DNA origami	$52\pm8^{*}$, $69\pm5^{**}$	single	52 ± 16	151 ± 31	22 ± 8	37 ± 11	60
		multi	53 ± 15	157 ± 30	22 ± 8	37 ± 10	60
Clathrin pits, 1:200	$100^{\dagger}$, $74\pm13^{\ddagger}$	single	86 ± 19	156 ± 23	57 ± 22	65 ± 20	43
		multi	86 ± 19	156 ± 23	57 ± 22	65 ± 20	43
Clathrin pits, 1:500	$100^{\dagger}$, $74\pm13^{\ddagger}$	single	87 ± 20	183 ± 68	57 ± 26	58 ± 17	30
		multi	91 ± 23	178 ± 56	54 ± 20	58 ± 17	30
Vaccinia virus particles	108 ± 29	single	117 ± 48	190 ± 81	56 ± 15	74 ± 57	11
		multi	137 ± 62	221 ± 135	58 ± 17	76 ± 61	11
Podosome cores		single	132 ± 19	230 ± 82	125 ± 46	206 ± 85	30***
		multi	128 ± 14	237 ± 60	128 ± 48	213 ± 87	30 ***
